# ANN trained by BBO for modeling of fly ash cementitious systems with high range water reducing admixtures

**DOI:** 10.1038/s41598-025-32972-1

**Published:** 2026-02-01

**Authors:** Naz Mardani, Ramin Kazemi, Murteda Unverdi, Ali Mardani, Seyedali Mirjalili

**Affiliations:** 1 Mathematics Education Department, Bursa Uludag University, Bursa, Turkey; 2 Independent Researcher, Sabzevar, Iran; 3 Civil Engineering Department, Bursa Uludag University, Bursa, Turkey; 4 Centre for Artificial Intelligence Research and Optimisation, Torrens University Australia, Brisbane, Australia; 5 Faculty of Electrical Engineering and Computer Science, VSB – Technical University of Ostrava, Ostrava, Czech Republic; 6 University Research and Innovation Center, Obuda University, Budapest, Hungary

**Keywords:** Cementitious systems, Fly ash, High-range water-reducing admixture, Artificial intelligence, Artificial neural network, Biogeography-based optimisation

## Abstract

This study aims to develop artificial intelligence (AI) models for predicting the compressive strength and flow value of cementitious systems containing fly ash, influenced by various high-range water-reducing admixtures (HRWRAs) that differ in molecular weight and chain length. A database comprising 180 mixes was created, encompassing cement and fly ash dosages, HRWRA characteristics (including molecular weight, main and side chain lengths) curing period, and flow time. Two AI-based modelling approaches were employed: a classical artificial neural network (ANN) and a new hybrid model that integrates ANN with biogeography-based optimisation (ANN–BBO). The modeling results showed that the hybrid model achieved a compressive strength performance with an R^2^ of approximately 0.99 and an RMSE of around 1.37 MPa, while the single ANN model attained an R^2^ of about 0.91 and an RMSE of 4.40 MPa. For flow value prediction, the ANN–BBO model also demonstrated high accuracy (R^2^ ≈ 0.98; RMSE ≈ 0.32 cm). Furthermore, the ANN–BBO model reduced the prediction error by approximately 60% across the evaluation criteria compared to the single ANN model, highlighting its enhanced performance. The importance of the input variables indicated that curing time and cement content have the greatest impact on compressive strength, while flow time and the molecular weight of the HRWRA significantly influence the flow value. Since AI models rely solely on virtual trials, they significantly reduce laboratory time and material usage while aiding in the design of mixes with lower water-to-binder ratios and higher fly ash content, which ultimately helps to reduce the CO_2_ footprint. The proposed models provide a practical route to low-clinker, FA-rich mix designs that satisfy strength/workability targets with less cement, supporting embodied-carbon reductions and straightforward integration into ready-mix/precast quality-control workflows.

**Supplementary Information:**

The online version contains supplementary material available at 10.1038/s41598-025-32972-1.

## Introduction

Water-reducing admixtures (WRAs) have become indispensable in contemporary concrete technology, as they enhance workability and durability while reducing the water content in cementitious mixtures. These admixtures contribute to improved concrete performance by enabling lower water-to-cement (w/c) ratios without adversely affecting workability. The optimization of w/c ratios is essential for attaining the desired properties of concrete, given its direct influence on the strength and durability of the final product. In recent developments, the design of advanced WRAs has increasingly focused on modifying their chemical architecture to enhance performance across diverse cementitious systems.

WRAs have undergone substantial advancements, with scholarly investigations demonstrating that the precise chemical formulation and architecture of these additives are critical in augmenting the characteristics of cementitious systems^1^. This persistent innovation seeks to optimize the functionality of WRAs, particularly by resolving compatibility challenges and enhancing their impact on both the properties of fresh and hardened concrete. The ongoing enhancement of these additives is imperative for satisfying the escalating requirements of contemporary construction methodologies^2^.

Cementitious systems play a pivotal role in construction, delivering the structural strength and stability (flow retention over time) required for various applications. The selection of constituent materials and their proportions significantly impacts both the mechanical properties and durability of these systems^3^. Beyond the mechanical aspects of concrete, carbon emissions have emerged as a major environmental concern^4,5^. In particular, in alignment with the European Green Deal, the construction sector is placing greater emphasis on sustainable approaches, such as incorporating supplementary materials like fly ash to improve concrete performance while simultaneously reducing its ecological footprint^6,7^.

Fly ash plays a crucial role in enhancing the sustainability of cementitious materials by lowering carbon emissions and improving mechanical performance, thereby promoting environmentally friendly construction practices^8^. This strategy aligns with the increasing focus on sustainable building materials that minimize the environmental impact of concrete production, as demonstrated by the notable advantages linked to the use of fly ash^9–11^. In concretes incorporating fly ash, considerations regarding workability and strength are of critical importance, since an appropriate proportion of fly ash can enhance performance without compromising durability^12^. Moreover, the integration of advanced WRAs with fly ash has the potential to produce optimized concrete mixtures that satisfy both structural performance and environmental sustainability criteria^13^. Current research continues to explore how various WRA chemical structures influence their interaction with fly ash, with the goal of further improving both the mechanical performance and environmental sustainability of concrete^14^.

The application of high-range water-reducing admixtures (HRWRAs) can markedly improve the mechanical performance of concrete, especially when used in conjunction with supplementary cementitious materials such as fly ash. This combination not only enhances workability but also significantly contributes to the long-term durability of concrete structures, increasing their resilience to environmental stressors^15^. The synergistic effect (combined effect greater than the sum of individual effects; positive interaction) of HRWRAs and fly ash not only optimizes concrete performance but also plays a vital role in decreasing the carbon emissions linked to cement production^16,17^. This reduction is particularly important considering the substantial share of the construction industry in global greenhouse gas emissions^18–21^.

A laboratory-based approach offers valuable insights into the compressive strength (CS) of concrete and the interaction mechanisms between HRWRAs and fly ash within cementitious matrices, thereby supporting the development of more environmentally friendly concrete alternatives^22^. However, the success of these formulations is highly dependent on the specific chemical structures of the HRWRAs employed, which can exhibit varying degrees of compatibility with fly ash. Consequently, laboratory experimentation may encounter several drawbacks, including time constraints, consumption of raw materials, variability in material properties, limited formulation scope, and the need for extensive data to comprehensively assess performance outcomes^23^.

To overcome these limitations, machine learning techniques are increasingly utilized to optimize the formulation of cementitious systems. These approaches enhance predictive capabilities and material efficiency, thereby contributing to more sustainable construction practices^24^. The integration of advanced artificial intelligence (AI) technologies into optimization processes allows for more accurate performance predictions and more efficient use of materials in concrete production^25^. These technologies not only improve prediction accuracy but also promote sustainability by minimizing waste and resource consumption in construction. Within these frameworks, algorithms evaluate the influence of various input parameters on construction outcomes, thus enhancing decision-making and operational efficiency. Through the application of machine learning, construction professionals can reduce project timelines and costs while maintaining specification-compliant performance in materials and design^26^.

Machine learning approaches, particularly artificial neural networks (ANN), have demonstrated substantial potential in forecasting the performance of cementitious systems by capturing complex interactions between input parameters and resulting properties. This predictive capability facilitates more informed decisions in concrete mixture design, ultimately contributing to improved sustainability and efficiency in construction practices^27,28^. Artificial intelligence technologies enhance design efficiency and broaden the scope for material exploration, surpassing the limitations of conventional methods. This advancement supports sustainable construction by optimizing resource use and minimizing material waste. The evolution of AI tools continues to boost productivity, reduce waste generation, and enable the development of tailored concrete formulations that meet specific project requirements^29^.

Recent studies underscore a growing focus on modeling cementitious systems, especially those incorporating fly ash. These studies highlight the vital importance of advanced modeling techniques in precisely assessing the mechanical behavior of such systems. Table 1 presents a summary of key AI-driven modeling research relevant to cementitious materials.

**Table 1:** AI-based modeling studies selected from the literature in civil engineering.

References	Product or waste material used in mixtures	Dataset	AI method	The best model for CS (R^2^ value)
^30^	HRWR	54	ANN-1, ANN-2	*ANN-1 (0.9431)
^31^	High volume fly ash	450	LR, NLR, ANN, M5P-tree,	*NLR (0.9969)
^32^	Phase change materials	154	RFR, ETR, GBR, XGBR,	*GBR (09,884)
^33^	Blast furnace slag, superplasticizer	1030	LSSVM-CSA, GP	*LSSVM-CSA (0.9767)
^34^	Fly ash	112	MPMR, RVM, ENN, GP, ELM	*MPMR (0.9920)
^35^	Fly ash	196	MLR, PSO-ANFIS, ANN, GA-ANFIS	*GA-ANFIS (0.9726)
^26^	Fly ash, blast furnace slag	1030	Bagging, GEP, AdaBoost, DT	*Bagging (0.9200)
^36^	Metakaolin	276	ANN, ANFIS	*ANFIS (0.9909)
^37^	Calcined sludge	2160	LR, SVM, RF, Ensemble, CNN, MLP-ANN	*CNN (0.9949)
^38^	Rice husk ash, superplasticizer, fly ash	93	GEP, ANN	*ANN (0.9695)
^39^	Fly ash, superplasticizer	100	GEP, ANN, DT, Bagging regressor	*Bagging regressor (0.9849)
^40^	RCA, fly ash, silica fume	380	SVM, ANN, AdaBoost, CNN	*CNN (0.9670)
^41^	Fly ash, superplasticizer	190	BP-ANN, RF, XGBoost, BP-GA, BP-PSO	*BP-GA (0.9731)
^42^	Fly ash, superplasticizer	130	FQ, IN, M5P-tree	*FQ (0.9849)
^43^	Fly ash	315	ANN, KNN, SVR, XGB	*ANN (0.9758)

*Represents the best model for CS.

Previous research has confirmed the effectiveness of various AI methods, particularly ANN, in predicting the CS of concrete mixtures containing fly ash and other supplementary materials, thereby demonstrating their potential to enhance performance and promote sustainability in construction practices^24,34^. Moreover, these techniques continue to advance, offering valuable insights into the optimization of cementitious systems to achieve improved environmental outcomes, ultimately contributing to the reduction of greenhouse gas emissions associated with concrete production and supporting sustainable construction initiatives^44,45^. The capacity of ANNs to identify and learn from complex data patterns enables more precise predictions of concrete behavior, thus fostering the development of innovative and environmentally friendly solutions within civil engineering applications^38,46^. In addition, ANNs facilitate the exploration of new concrete formulations aligned with sustainability objectives, thereby improving both the efficiency and ecological performance of construction projects^47,48^.

The limitations of current AI methodologies are often linked to their dependence on large datasets and the inherent difficulties in obtaining high-quality, representative data for training. Moreover, insufficient data availability can impair the predictive capabilities of machine learning models in evaluating concrete performance. Many AI techniques, particularly deep learning neural networks, face significant challenges when confronted with limited data, emphasizing the need to explore alternative strategies that maintain predictive reliability. Ongoing research seeks to enhance the robustness of AI models by incorporating transfer learning and data augmentation techniques, which can improve prediction accuracy and model generalization under data-constrained conditions^11^. Effective AI integration in engineering requires expertise across both AI and civil engineering domains, along with a collaborative approach to overcome the complex challenges faced in the construction industry. Highlighting the importance of interdisciplinary knowledge is essential for the successful application of AI technologies in sustainable construction practices^24,49,50^.

Researchers are actively exploring innovative techniques, such as synthetic data generation and transfer learning, to enhance data acquisition and model training in light of these existing limitations. Additionally, the use of binary AI models improves data interpretability for engineers, thereby strengthening decision-making and model resilience in complex real-world conditions and expanding the scope of AI applications in civil engineering projects^19,41^.

**Fig. 1:**
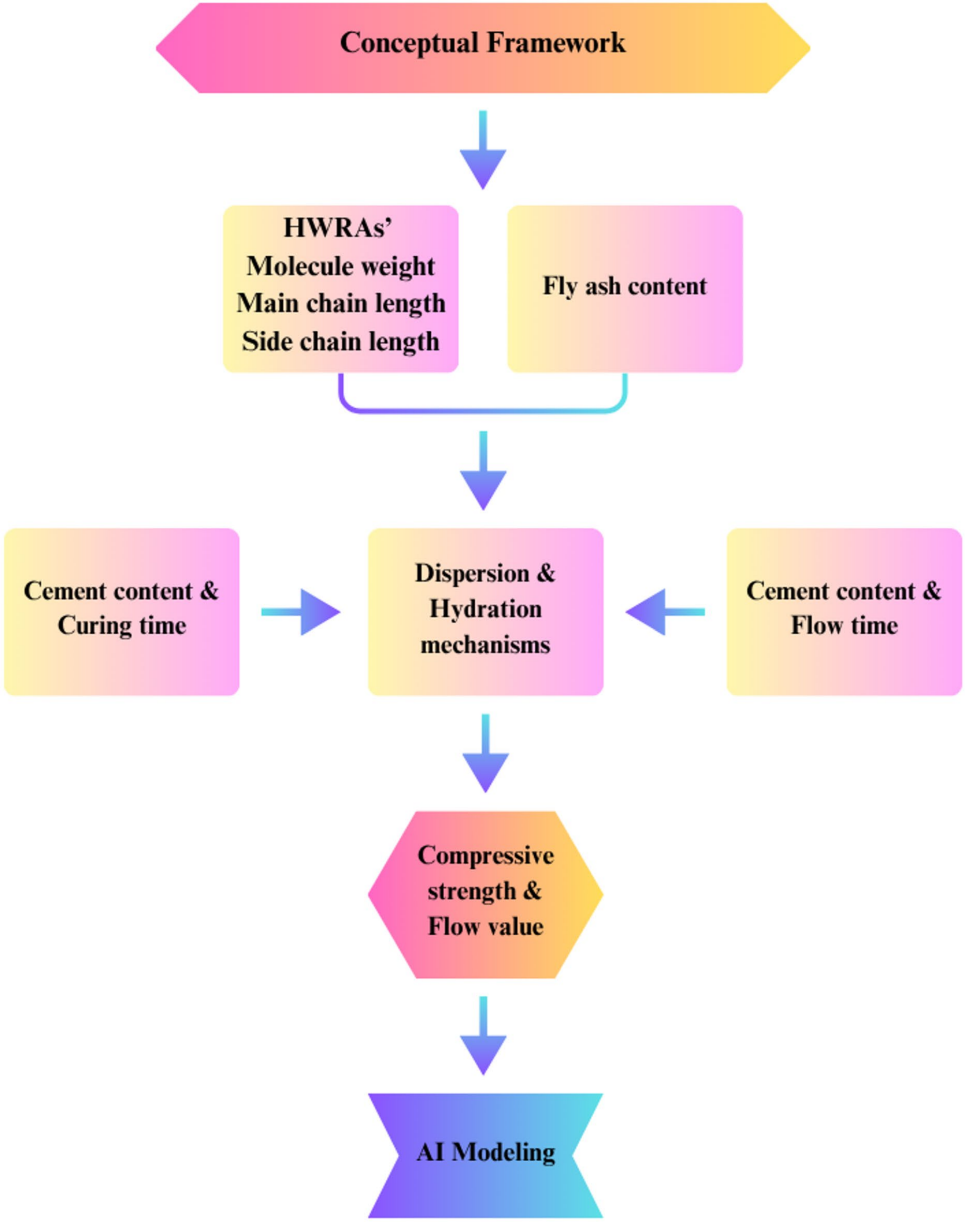
Conceptual framework of the current study.

In the modeling of cementitious systems, most proposed models function in isolation, and hybrid approaches integrating meta-heuristic optimization techniques have yet to be comprehensively explored. Furthermore, a review of current modeling studies reveals a noticeable gap in research focusing on the flow value (FV) of cement mortars, a critical parameter for evaluating the workability and overall performance of cementitious mixtures. Considering these identified gaps, there is a clear need for comprehensive optimization strategies and innovative modeling approaches that can effectively address the FV of cement mortars and improve the predictive performance of ANN techniques within cementitious systems. The integration of ANN with biogeography-based optimization (BBO) presents a novel methodology aimed at enhancing predictive accuracy by optimizing neural network parameters. This combined approach not only increases the precision of performance predictions but also contributes to the formulation of more efficient and sustainable concrete mixtures for civil engineering applications^29^. The current absence of the ANN–BBO model in studies concerning cementitious systems will be addressed in this research, thereby improving the analysis of FV and enriching the literature on flow value modeling. Figure 1. depicts the conceptual framework of the current study.

**Fig. 2:**
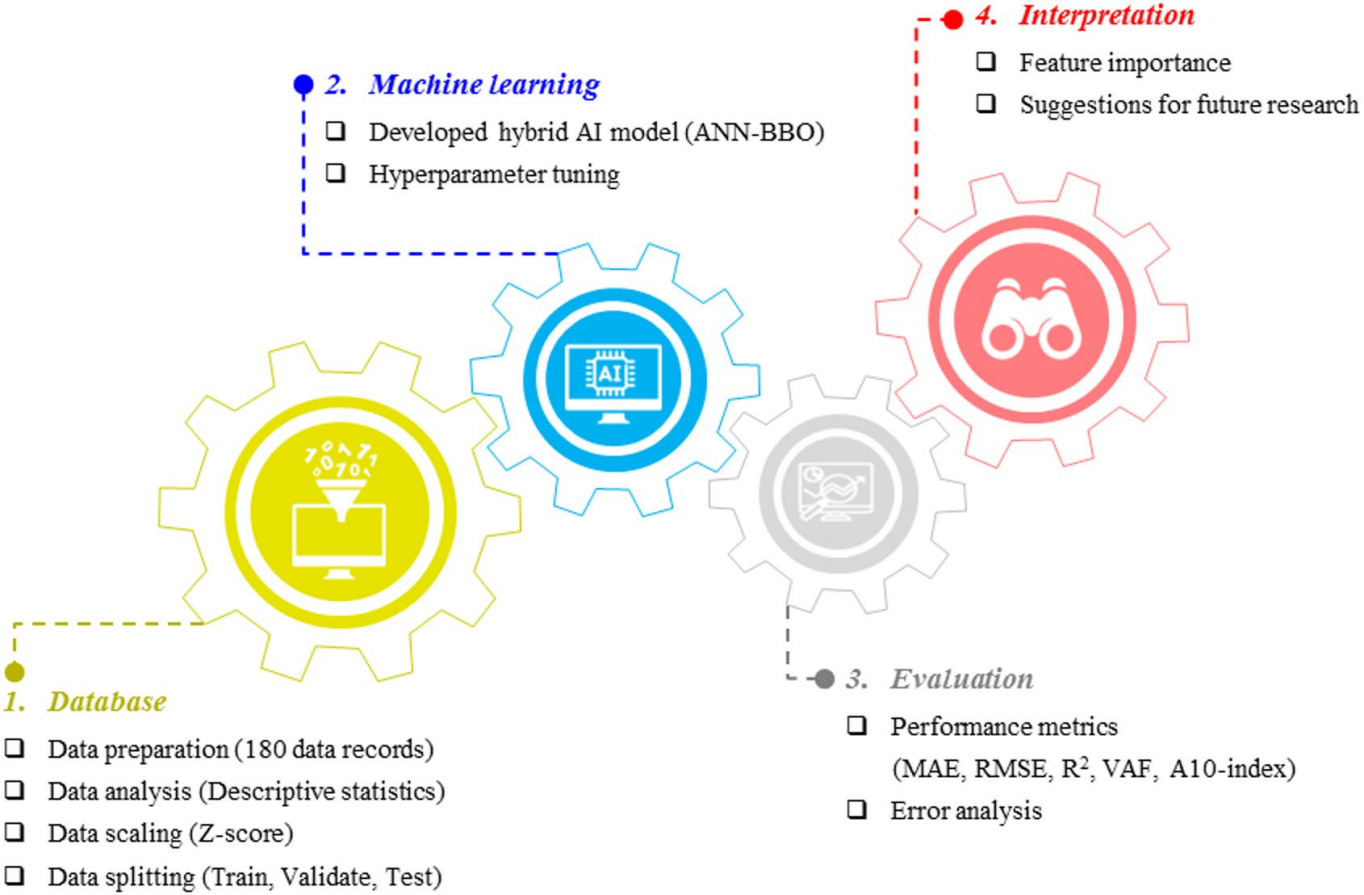
Research process in this study.

In this study, the use of ANN, along with its integration with the BBO algorithm, is expected to yield notable advancements in predicting both the CS and FV of cementitious systems, ultimately supporting sustainable construction efforts. The data utilized in this research were obtained from three studies examining the influence of side and main chain lengths of HRWRAs in fly ash-based cementitious systems and were used as input for the modeling process. This study aims to assess the predictive performance and engineering relevance of the proposed models. The results are anticipated to expand existing knowledge and offer practical insights into improving cement mortar properties. Ultimately, the research will highlight the benefits of integrating artificial intelligence with optimization techniques for advancing effective applications in civil engineering. An overview of the research framework proposed in this study is illustrated in Fig. 2.

Conventional prediction methods in cementitious systems -w/c-based rules, semi-empirical models, and low-order regressions- struggle to represent nonlinear, time-dependent, and structure–property effects arising from HRWRA molecular architecture and FA replacement. Our AI approach (ANN and ANN–BBO) learns these interactions directly from data, improving accuracy and enabling fast “what-if” optimization of strength–flow–cement content, which traditional tools cannot reliably deliver.

## Experimental dataset, variables description and mix design

This study explores the predictive modeling of CS and FV in fly ash-blended cementitious mortars incorporating HRWRAs, utilizing a dataset comprising 180 distinct mortar mixtures. The dataset was derived from a series of comprehensive and systematically executed experimental investigations^13,14,51^, which examined the molecular characteristics of HRWRAs, specifically the influence of main chain length, side chain length, and molecular weight, on the fresh and hardened properties of cementitious systems.

### Materials and admixture characteristics

The experimental program employed CEM I 42.5R Portland cement, Class F fly ash, standardized quartz sand as the fine aggregate, and three different polycarboxylate ether (PCE)-based HRWRAs. The physical, mechanical, and chemical properties of the cement and fly ash used in the experimental and modeling studies are presented in Table 2. ^13,14,51^. The distinguishing parameters among the HRWRAs were their molecular weights (kg/mol), main chain lengths, and side chain lengths (g/mol). This controlled variation enabled a focused examination of the influence of molecular architecture on the performance of the cementitious systems.

**Table 2:** Chemical composition, physical and mechanical properties of cement and fly ash.

Item	Amount (%)	
	Cement	Fly ash
SiO_2_	18.86	59.22
Al_2_O_3_	5.71	22.86
Fe_2_O_3_	3.09	6.31
CaO	62.7	3.09
MgO	1.16	1.31
SO_3_	2.39	0.17
Na_2_O + 0.658 K_2_O	0.92	1.4
Cl^−^	0.01	0.001
Insoluble residue	0.32	0.32
Loss on ignition	3.2	3.2
Free CaO	1.26	0
Physical properties		
Specific gravity	3.15	2.31
Blaine specific surface (cm^2^/g)	3,530	4,300
Residual on 0.045 mm sieve (%)	7.6	10
Mechanical properties		
Compressive strength (MPa)		
1-day	14.7	-
2-day	26.8	-
7-day	49.8	85.9
28-day	58.5	100.7
90-day	-	110.2

### Mortar mix design and proportions

A range of cement mortars were prepared, incorporating different admixture molecular structures and fly ash replacement rates of 0%, 15%, 30%, and 45% by total cement weight. All mixtures were produced with a constant w/c ratio of 0.485 and a sand-to-binder ratio of 2.75, following the specifications of ASTM C109.

### Sample preparation and curing

The mortars were mixed and cast under controlled laboratory conditions to guarantee uniformity. Following casting, specimens were compacted using standardized procedures and demolded after 24 h. Subsequently, curing was performed in lime-saturated water at 23 ± 2 °C, adhering to conventional curing protocols to replicate typical field conditions.

### Testing of fresh and hardened properties

Flow properties were measured in accordance with ASTM C1437-20, evaluating the time-dependent spread behavior of the mortar mixtures. Compressive strength tests were conducted at various curing intervals (1, 3, 7, 28, and 90 days) following the ASTM C109 standard. Each testing protocol was replicated across different admixture formulations and fly ash replacement levels to ensure the reliability and statistical significance of the results.

### Definition of input and output variables

The experimental results were carefully recorded. The key parameters considered in this study included the amount of cement (*X*_1_), fly ash (*X*_2_), molecular weight of the HRWRA (*X*_3_), main chain length of the HRWRA (*X*_4_), side chain length of the HRWRA (*X*_5_), curing time (*X*_6_), and flow time (*X*_7_), all of which were rigorously analyzed and treated as input variables influencing the overall performance of the mortar mixture. Conversely, the variables related to performance outcomes, specifically CS (*Y*_1_) and FV (*Y*_2_), were systematically classified as dependent output variables, expected to quantitatively reflect the effects of the aforementioned input parameters. This organized categorization of variables enabled precise modeling of the relationships among admixture properties, mix design parameters, and mortar performance indicators.

### Data normalization and statistical processing

**Fig. 3:**
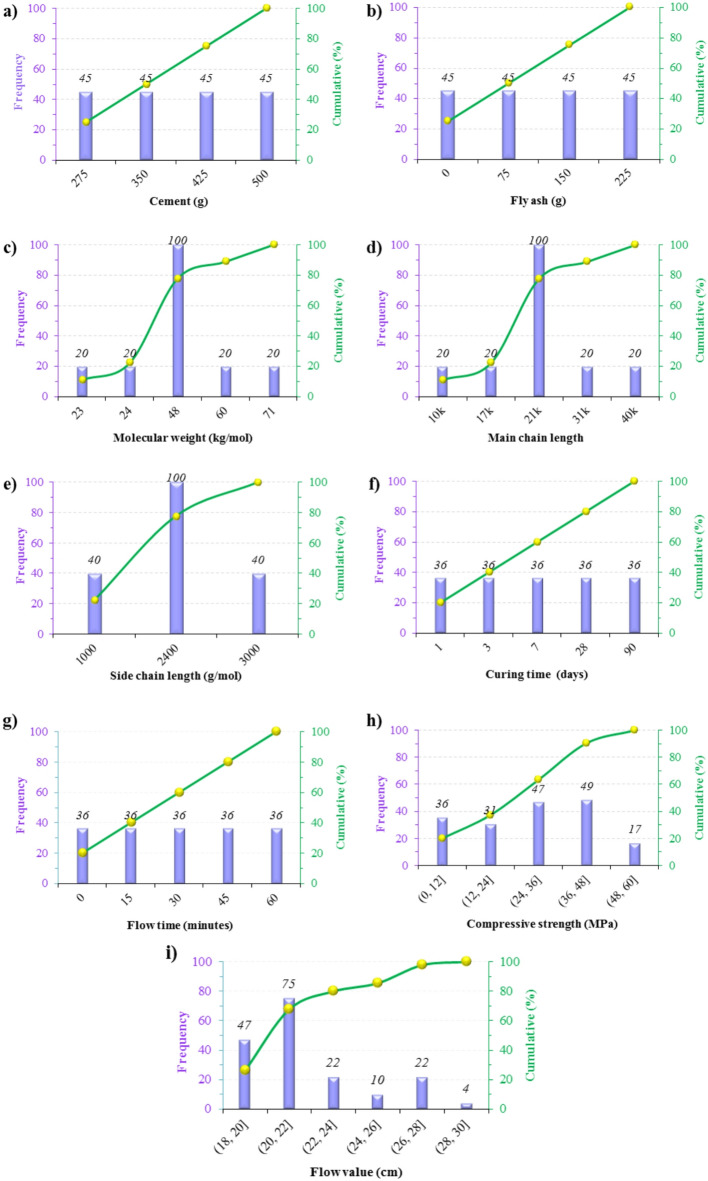
Histograms of the frequency of variables: inputs (a-g) and outputs (h & i).

For machine learning applications, all variables were normalized using the Z-score standardization method. This procedure eliminated dimensional biases and facilitated the convergence of predictive algorithms. Figure 3 depicts the histograms of the frequency distribution of the variables. Furthermore, key descriptive statistics (including the mean, standard deviation, kurtosis, and skewness) are presented in Table 3 to assess the distribution and variability of each variable in the dataset.

**Table 3:** Descriptive statistics of the variables.

ID No.: variables	Category	Unit	Range	Mean	Standard deviation	Kurtosis	Skewness
*X*_1_: Cement	Input for *Y*_1_ & *Y*_2_	g	275–500	387.50	83.85	-1.36	0.00
*X*_2_: Fly ash	Input for *Y*_1_ & *Y*_2_	g	0–225	112.50	83.85	-1.36	0.00
*X*_3_: Molecular weight	Input for *Y*_1_ & *Y*_2_	kg/mol	23–71	46.44	14.35	-0.48	-0.26
*X*_4_: Main chain length	Input for *Y*_1_ & *Y*_2_	-	10–40	22.56	8.00	0.39	0.82
*X*_5_: Side chain length	Input for *Y*_1_ & *Y*_2_	g/mol	1000–3000	2222.22	695.67	-0.51	-0.89
*X*_6_: Curing time	Input for *Y*_1_	day	1–90	25.80	33.51	-0.15	1.24
*X*_7_: Flow time	Input for *Y*_2_	minute	0–60	30.00	21.21	-1.303	0.00
*Y*_*1*_: Compressive strength	Output of *Y*_1_	MPa	1.8–57.7	21.967	2.463	0.280	1.133
*Y*_*2*_: Flow value	Output of *Y*_2_	cm	18.3–28.5	21.968	2.607	-0.089	1.007

Outlier detection and treatment were carefully conducted prior to model development. Initially, the dataset was screened using both statistical (z-score analyse) and visual methods (Histogram plots) to identify abnormal values. Observations with unrealistic or inconsistent measurements were cross-checked against the original laboratory records, and only those confirmed as experimental errors were removed. All valid data points representing natural variability in material behavior were retained to preserve the representativeness of the dataset.

This systematic and scientifically rigorous data preparation established a robust foundation for the subsequent implementation of both ANN and ANN–BBO hybrid models. The insights derived from this process contribute to the advancement of high-performance, sustainable cementitious systems optimized through AI.

### Selection of influencing variables

The input set was chosen to (i) be available at mix-design time, (ii) maintain a clear mechanistic link to strength and workability in FA–HRWRA systems, and (iii) avoid confounding with controlled factors. Variables were standardized prior to modeling.

- X_1_: Cement—Principal contributor of clinker and early hydration products; governs paste reactivity and is a primary driver of compressive strength and water demand.
- X_2_: Fly ash (FA)—Modulates the dilution–pozzolanic balance and particle packing; at fixed w/c, higher FA typically lowers early strength but can improve later-age strength and influences flow via particle morphology and glass content. Modeling Cement and FA separately (rather than as a single “binder%”) preserves their counter-varying effects.
- X_3_: Molecular weight (HRWRA)—Encodes hydrodynamic size/distribution of the PCE and relates to adsorption layer thickness and dispersion efficiency, affecting flow and early paste structure.
- X_4_: Main chain length—Proxies backbone anchoring/adsorption on cement surfaces and competitive adsorption under changing ionic strength; impacts steric stabilization and dispersion robustness.
- X_5_: Side chain length—Governs steric hindrance and flow retention; longer side chains generally mitigate re-flocculation and improve time-dependent workability.
- X_6_: Curing time—Captures hydration progress and secondary reactions (including FA contribution), the dominant driver of compressive strength evolution.
- X_7_: Flow time—Captures thixotropy/structuration and HRWRA retention effects, the dominant driver of flow evolution.

The water-to-cement ratio (w/c), sand ratio, temperature, and mixing protocol were held constant in the experimental program and therefore not modeled to avoid confounding. HRWRA dosage was fixed within series; consequently, the analysis emphasizes architecture descriptors (X_3_–X_5_) as the differentiating admixture factors.

Because Cement and FA sum to the binder and can be collinear, both were retained for interpretability and monitored via diagnostic checks (e.g., feature-importance stability and sensitivity analyses). Resulting importances were consistent with mechanism: Curing time and Cement/FA dominate compressive strength, whereas Flow time and Side chain length most strongly influence flow, with Molecular weight and Main chain length providing secondary but meaningful contributions.

These choices reflect practical levers available to mix-design engineers and align with the controlled domain of this study (Class F FA; PCE-type HRWRA). Extensions to variable w/c, broader admixture families, and multi-lab conditions are reserved for future work. The complete mix desing is provided in Supplementary Table S1.

## Overview of the AI methods employed

### Artificial neural network (ANN)

**Fig. 4:**
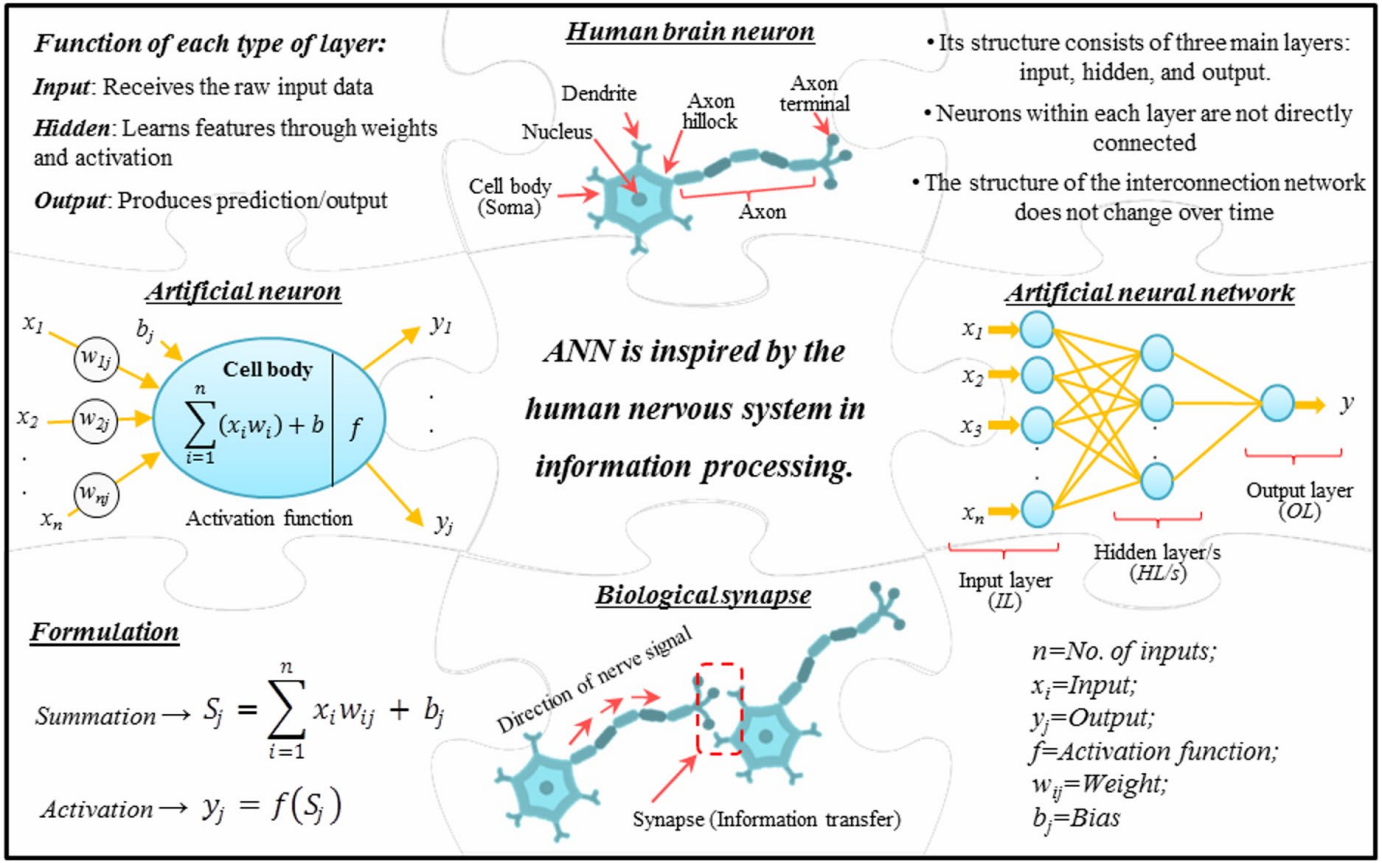
A schematic illustrating the introduction and performance of the ANN model.

The first attempt to develop a mathematical model of an ANN was undertaken by McCulloch and Pitts^52^. The concept of ANNs was inspired by the structure and function of biological neural networks found in the human brain. The computational structure of ANNs are modeled on the signal transmission mechanisms observed in nerve cells (neurons) in the human brain. They simulate the process of receiving input signals and producing corresponding outputs through interconnected layers of artificial neurons. An ANN is generally composed of three primary layers:

- An input layer (*IL*), which receives data and signals from outside sources, with a neuron count equal to the number of input variables;
- An output layer (*OL*), which delivers the final predictions or outcomes of the model, with its neurons representing the number of output variables of the modeled problem; and
- Hidden layer/s (*HL/s*) positioned between layers of *IL* and *OL*, handle the internal processing of information within the network. The number of neurons in these *HL/s* can vary, and determining an optimal number is crucial for ensuring high model accuracy^53,54^. At a glance, Fig. 4 provides an overview of the ANN model inspired by the human nervous system and its structure and function.

### Biogeography-based optimization (BBO)

In 2008, BBO was developed by Simon^55^ as an evolutionary algorithm inspired by the concept of biogeography. Biogeography deals with how species evolve from their distribution in ecosystems to their displacement and extinction, where habitats are small ecosystems composed of diverse populations of creatures that are typically segregated from other habitats. Regions that can support a diverse range of creatures are evaluated using a habitat suitability index (HSI). The HSI of a given habitat is influenced by characteristics such as climate, vegetation, etc. These characteristics, known as suitability index variables (SIVs), play a key role in determining the HSI. A habitat with a high HSI suggests it offers suitable conditions for supporting larger populations, whereas a low HSI reflects poor conditions that often lead to increased migration and population decrease. Consequently, regions with high HSI typically exchange SIV with those having low HSI. The two main operators considered in this optimization method are:

- Migration: This process facilitates the finding of new regions in the search space by switching solutions between habitats through emigration and immigration rates to enable global search capabilities.
- Mutation: By creating random conditions in the solution search, it not only increases the diversity in the population but also prevents stagnation in local optima.

**Fig. 5:**
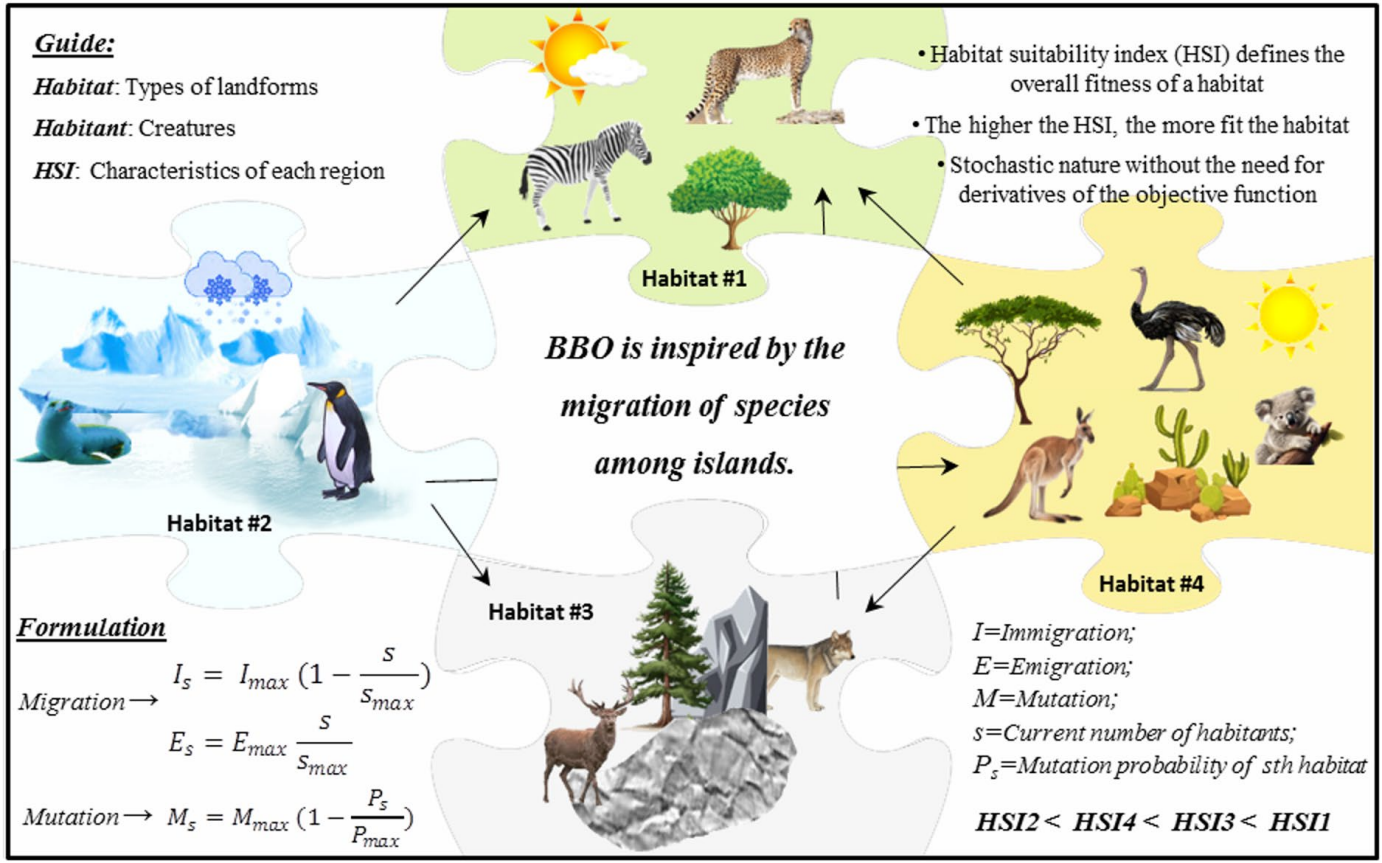
A schematic illustrating the introduction and performance of the BBO model.

Figure 5 provides an overview of the BBO method inspired by the principles of biogeography and its mathematical formulation.

After gaining a comprehensive understanding of the BBO algorithm, its distinctive advantages over other optimization methods become evident. The superior performance of BBO primarily arises from its unique migration and mutation mechanisms, which enable the algorithm to effectively escape local minima and maintain population diversity^56^. These operators introduce abrupt yet beneficial perturbations in the candidate solutions, thereby strengthening the algorithm’s exploration capability. In a comparative study by Mirjalili et al.^56^ the performance of BBO was evaluated against several established algorithms, including particle swarm optimization (PSO), genetic algorithm (GA), ant colony optimization (ACO), evolutionary strategies, and probability-based incremental learning. The findings demonstrated that, unlike these methods, BBO incorporates operators that foster sudden transitions in candidate solutions, reducing the likelihood of premature convergence to local optima. Furthermore, BBO exhibits desirable characteristics such as simplicity, flexibility, and computational efficiency, and, due to its stochastic, derivative-free nature, it can handle complex optimization problems without requiring gradient information. Previous research has confirmed that BBO is a robust and competitive algorithm capable of addressing a broad range of real-world engineering and scientific optimization challenges^57^.

## Model development

This section aims to provide a comprehensive description of the model development carried out to propose an AI approach for predicting the CS and FV of cementitious systems incorporating fly ash. To accomplish this, two AI modeling strategies are implemented: a classical ANN and a hybrid model that integrates ANN with BBO. The comparison between these models provides important insights into the performance of the hybrid approach relative to the classical model. The metaheuristic optimization method of BBO is used to determine the optimal parameters, aiming to improve the prediction model’s accuracy and reduce errors. Data preprocessing is known as a crucial early step in the modeling process, as it greatly enhances data quality by eliminating biases resulting from differences in scale or units among variables. This, in turn, enhances the performance of the developed models. Accordingly, all data were normalized utilizing the Z-score method, as shown in Eq. (1).

1 $$Z=\frac{X- \mu }{\sigma }$$

Here, *X* represents the value of each variable, while *μ* and *σ* denote the mean and standard deviation of the data for that variable, respectively.

**Fig. 6:**
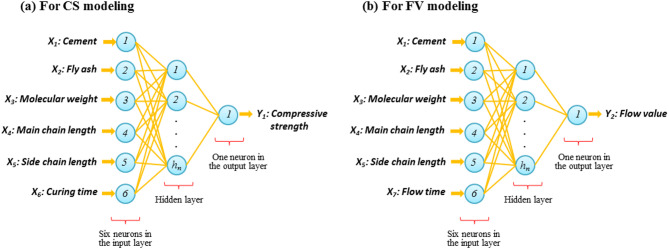
Model architecture for predicting (**a**) compressive strength and (**b**) flow value of cementitious systems containing fly ash.

When evaluating AI models, an important consideration is whether the developed model is the optimal choice within its hypothesis space, especially regarding its ability to generalize to new data. This issue is closely linked to how the data is divided, which is a vital aspect in the model development process. To ensure proper model generalization and prevent biased performance estimation, the present study employed a three-way holdout validation strategy consisting of separate training, validation, and testing datasets^58^. This approach minimizes the risk of over-optimistic results that may arise when the same dataset is repeatedly used for both model development and evaluation. According to Mehlig^59^, an effective data split involves three distinct subsets: (i) a training set to build the model and capture underlying data patterns; (ii) a validation set to fine-tune the model and control overfitting; and (iii) a testing set to independently assess generalization capability. Because model performance can be highly sensitive to the composition of training data, the available parameters were sampled across a broad range within each subset. Consequently, the complete dataset—comprising 180 records for both compressive strength (CS) and flow value (FV)—was randomly divided into training (134 records), validation (23 records), and testing (23 records) subsets to ensure an unbiased and statistically representative evaluation of the proposed models. Numerous studies evaluating various learning algorithms have reported that the Levenberg–Marquardt algorithm outperforms others^60^. Therefore, it has been selected for use in this study. Moreover, the hyperbolic tangent is employed as the transfer function, as outlined in Haykin’s highly cited book^61^. The architecture of the model, particularly the number of hidden layers (HL), affects the effective convergence of the model in the training process^62^. Based on the universal approximation theorem^59^, the models are designed with a single HL. Accordingly, the initial architecture of the models used for CS and FV prediction is structured as *6*-*HL*_neurons_-*1*, as illustrated in Fig. 6. To establish the final architecture of the model (i.e., *HL*_neurons_), a detailed analysis will be conducted at the beginning of the results section.

## Evaluation criteria

To evaluate the performance of the proposed models and determine the most effective one, various statistical metrics have been utilized. Table 4 provides a concise description of the metrics along with their mathematical formula and ideal values.

**Table 4:** Summary of statistical metrics used.

Metric formula		Brief description	Range ideal value
*Mean absolute error (MAE)*		The average magnitude of the errors between the predicted and actual values, regardless of their direction	(0, $+\infty$) 0
$$MAE=\frac{1}{N}\sum_{\mathrm{i}=1}^{N}\left|{A}_{i}-{{P}_{p}}_{i}\right|$$			
*Root mean squared error (RMSE)*		The measure of how widely errors are dispersed around the line of best fit	(0, $+\infty$) 0
$$RMSE=\sqrt{\frac{1}{N}\sum_{\mathrm{i}=1}^{N}{({A}_{i}-{P}_{i})}^{2}}$$			
*Coefficient of determination (R*^2^*)*		The metric used to evaluate how well the model explains the variability of the response variable	(0, 1) 1
$${{R}^{2}=\left(\frac{{\sum }_{\mathrm{i}=1}^{N}\left({A}_{i}-\overline{A }\right)\left({P}_{i}-\overline{P }\right)}{\sqrt{\left[{\sum }_{\mathrm{i}=1}^{N}{\left({A}_{i}-\overline{A }\right)}^{2}\right]\left[{\sum }_{\mathrm{i}=1}^{N}{\left({P}_{i}-\overline{P }\right)}^{2}\right]}}\right)}^{2}$$			
*Nash–Sutcliffe efficiency (NSE)*		Measures how closely a model’s predictions align with the actual values by comparing the relative magnitude of the prediction error variance to the variance of the actual values	($-\infty$, 1) 1
$$NSE=1-\frac{{\sum }_{\mathrm{i}=1}^{N}{\left({A}_{i}-{P}_{i}\right)}^{2}}{{\sum }_{\mathrm{i}=1}^{N}{\left({A}_{i}-\overline{A }\right)}^{2}}$$			
*Variance Accounted For (VAF)*		The metric to describe the variability of the dataset by the model	(0, 100) 100%
$$VAF=\left(1- \frac{var(A- P)}{var(A)}\right) \times 100$$			
*A10-index*		The proportion of data records whose predicted values deviate by ± 10% from the actual values	(0, 1) 1
$\frac{\mathrm{m}10}{N}$	$\mathrm{where}\; \mathrm{m}10=\mathrm{No}.\text{ of data with}$		
	$$0.90\le A/P\le 1.10$$		

The number of data related to *N* = total, *N*_*Tr*_ = training, *N*_*Va*_ = validating, and *N*_*Te*_ = testing sets. ${A}_{i}$ and ${P}_{i}$ = The actual and predicted values of the *i*th data, respectively. $\overline{A }$ and $\overline{P }$ = The average of total ${A}_{i}$ and ${P}_{i}$, respectively.

## Results and discussion

### Establishing the final architecture of the models

As stated in the model development section, identifying the appropriate number of *HL*_neurons_ and finalizing the model architecture involves thorough analysis. To do this, models with different architectures ranging from 10 to 20 *HL*_neurons_ are assessed. To guaranty a fair comparison, the models are run using identical subsets. The evaluation criteria R^2^ and RMSE are calculated for the models and ranked according to their performance results. The ranking method is that the top is assigned to the highest R^2^ and the lowest RMSE. Ultimately, the model that achieves the top overall ranking score is regarded as the best model. Table 5 presents the results of this analysis. According to these results, the model containing 16 *HL*_neurons_ achieves the top ranking score, indicating its superior performance over other *HL*_neurons_ architectures. Therefore, the model with the final architecture of *6*-*16*-*1* is selected.

**Table 5:** Model performance and rankings based on varying numbers of *HL*_neurons_.

No. of *HL*_*neurons*_	Evaluation criteria values				Ranking of criteria				Overall ranking score
	Compressive strength		Flow value		Compressive strength		Flow value		
	R^2^	RMSE	R^2^	RMSE	R^2^	RMSE	R^2^	RMSE	
*HL*_10_	0.953	1.485	0.931	0.412	2	3	2	1	8
*HL*_11_	0.966	1.463	0.947	0.385	3	4	3	2	12
*HL*_12_	0.972	1.417	0.961	0.376	6	6	5	4	21
*HL*_13_	0.978	1.404	0.970	0.355	7	7	7	6	27
*HL*_14_	0.980	1.396	0.975	0.344	8	8	8	8	32
*HL*_15_	0.988	1.372	0.978	0.320	10	10	9	11	40
*HL*_16_	0.991	1.365	0.986	0.325	11	11	11	10	43
*HL*_17_	0.985	1.375	0.982	0.334	9	9	10	9	37
*HL*_18_	0.970	1.422	0.968	0.349	5	5	6	7	23
*HL*_19_	0.967	1.488	0.957	0.360	4	2	4	5	15
*HL*_20_	0.945	1.525	0.923	0.384	1	1	1	3	6

Table 6 outlines the parameter settings used to enhance the clarity of the presented information. All model developments were carried out using MATLAB software.

**Table 6:** Settings of the parameters used.

Model	Parameter	Setting
ANN	Dataset segmentation	
	Training set	134 data
	Validation set	23 data
	Testing set	23 data
	No. of input parameter	6
	No. of hidden layer/nodes	1/16
	Learning algorithm	Levenberg–Marquardt
	Transfer function	Hyperbolic tangent
	Max. no. of epochs	500
BBO	Population size	100
	Max emigration rate	1
	Habitat modification	1
	Mutation probability	0.005

### Evaluating the performance of models

**Fig. 7:**
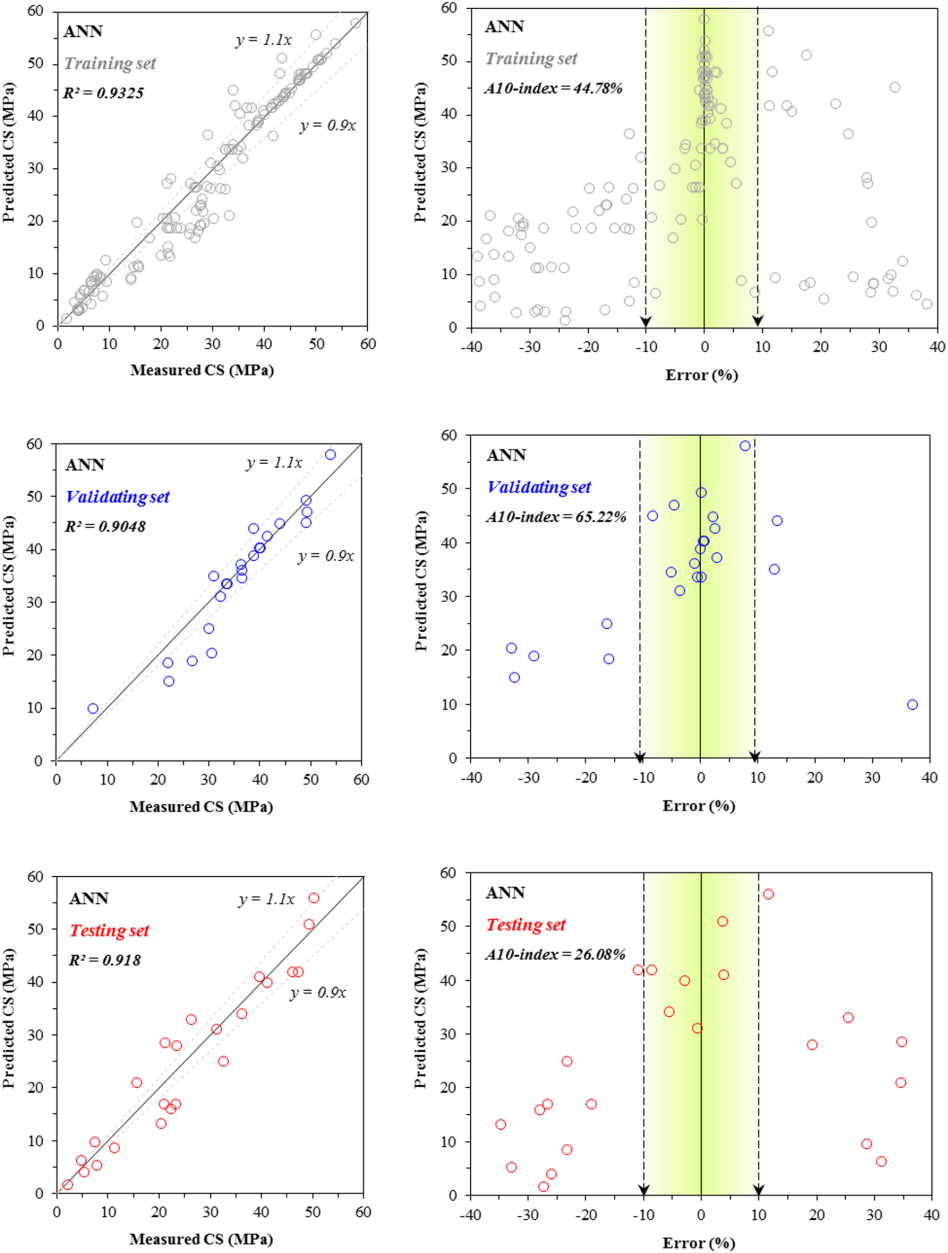
Comparison between the measured and predicted ANN values for CS, including the error distribution.

**Fig. 8:**
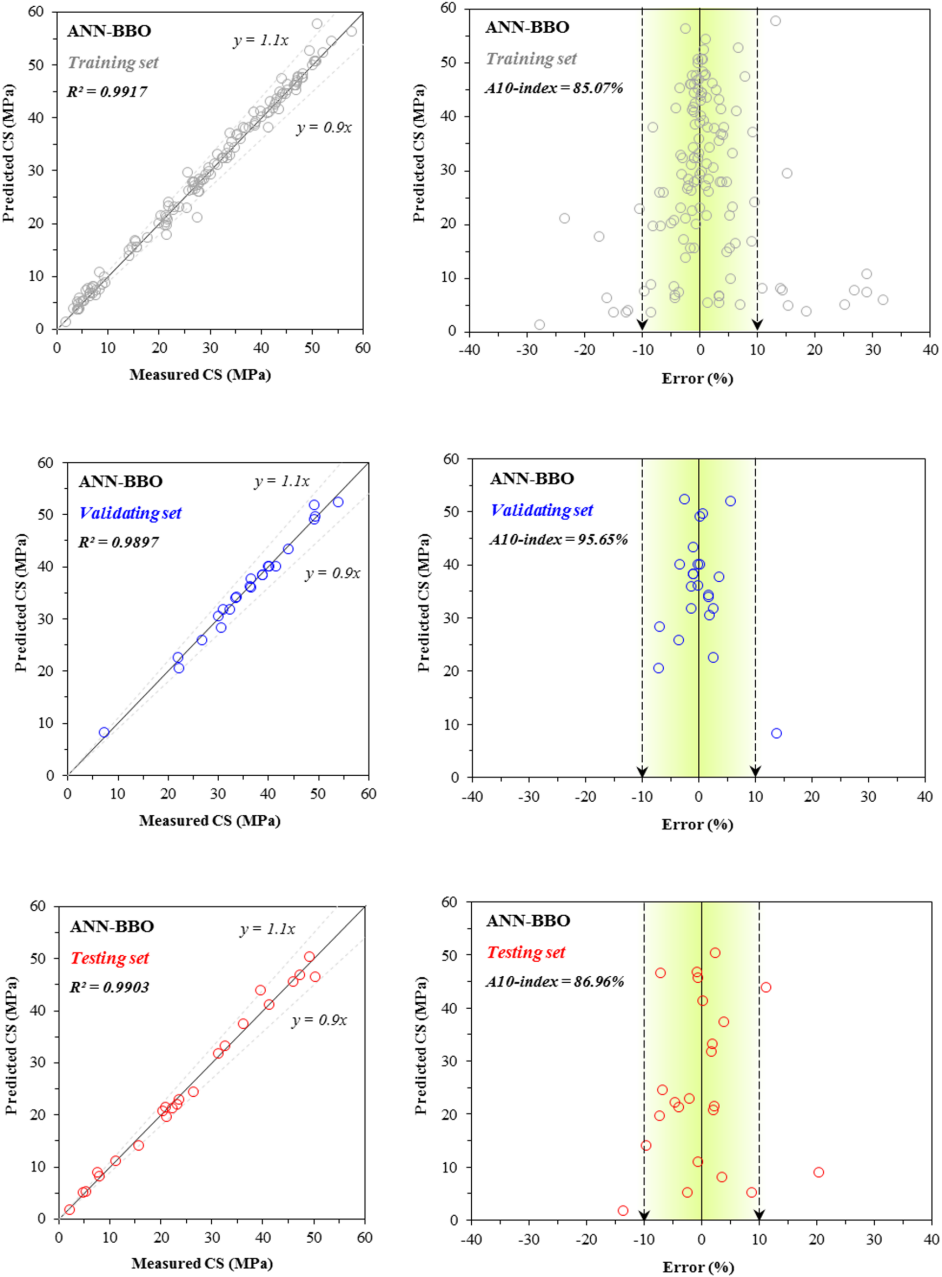
Comparison between the measured and predicted ANN–BBO values for CS, including the error distribution.

Figures 7 and 8 present the scatter plots and error distribution plots of CS modeling for the training, validating, and testing sets using the two developed models: ANN and ANN–BBO, respectively. The ANN model attained R^2^ values of 0.9325, 0.9048, and 0.9180 for the training, validating, and testing sets, respectively. In comparison, the ANN–BBO model achieved higher R^2^ values of 0.9917, 0.9897, and 0.9903 for the same sets. The results depict a stronger correlation between the measured and predicted values in the ANN–BBO model, with its data points aligning more closely with the ideal regression line (*y* = *x*) compared to those of the ANN model. To further assess the models, the figures illustrate the error distribution for each model across all three performance sets. A detailed analysis of the error margins reveals that for the ANN–BBO model, 85%, 95%, and 87% of predictions in the training, validating, and testing sets, respectively, fell within an error range of [-10%, 10%]. In comparison, the ANN model shows lower percentages of 44%, 65%, and 26% for the same sets. This clearly demonstrates the enhanced accuracy of the ANN–BBO model in predicting the CS of cementitious systems incorporating fly ash within a tighter error margin.

**Fig. 9:**
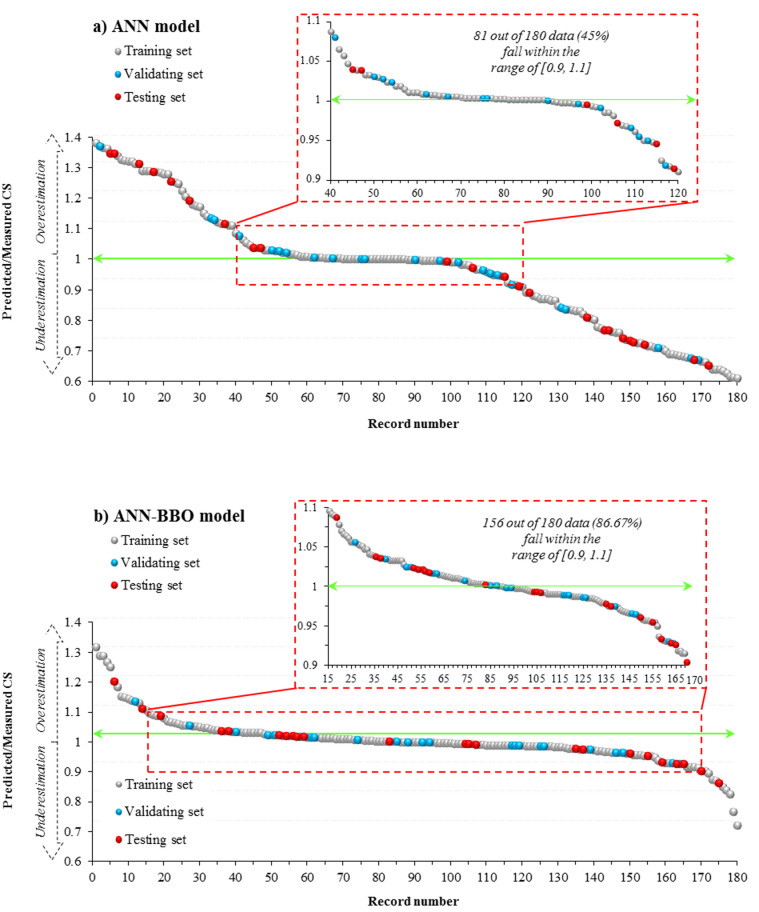
Ratios of predicted/measured CS value for the models (**a**) ANN and (**b**) ANN–BBO.

To better understand the models’ performance, Fig. 9 presents the ratios of predicted to measured CS values, offering a clear view of the model outputs for each data record in both developed models. Values on the vertical axis that are closer to 1 are considered desirable, as they reflect a strong similarity between the model predictions and the measured data. Figure 9 shows that the CS values predicted by the proposed ANN–BBO model align more closely with the measured CS values compared to those predicted by the classical ANN model. Specifically, 156 out of 180 data records (87%) fall within the 0.9–1.1 range for the hybrid ANN–BBO model, whereas only 81 out of 180 data records (45%) fall within this range for the single ANN model.

**Fig. 10:**
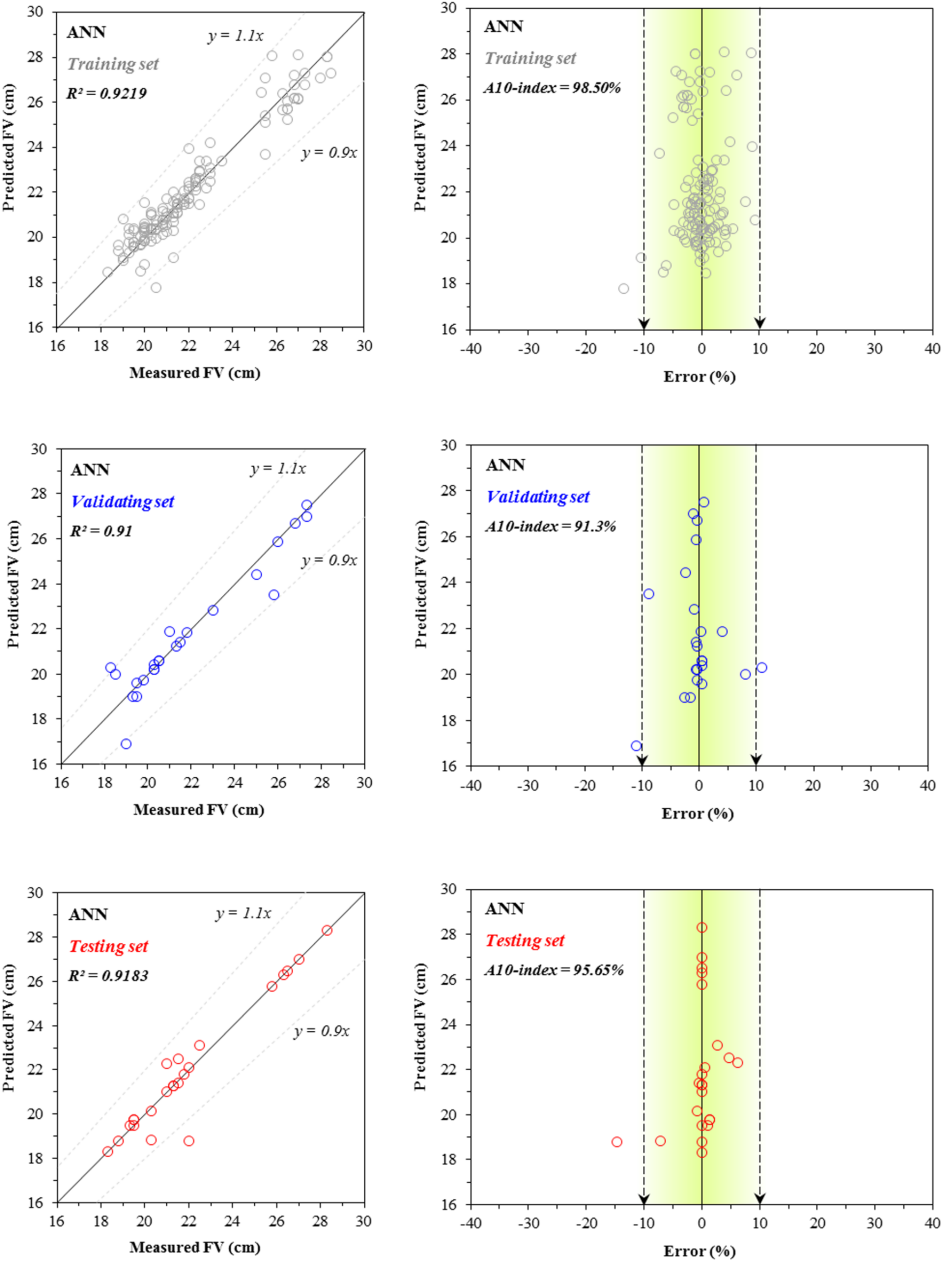
Comparison between the measured and predicted ANN values for FV, including the error distribution.

**Fig. 11:**
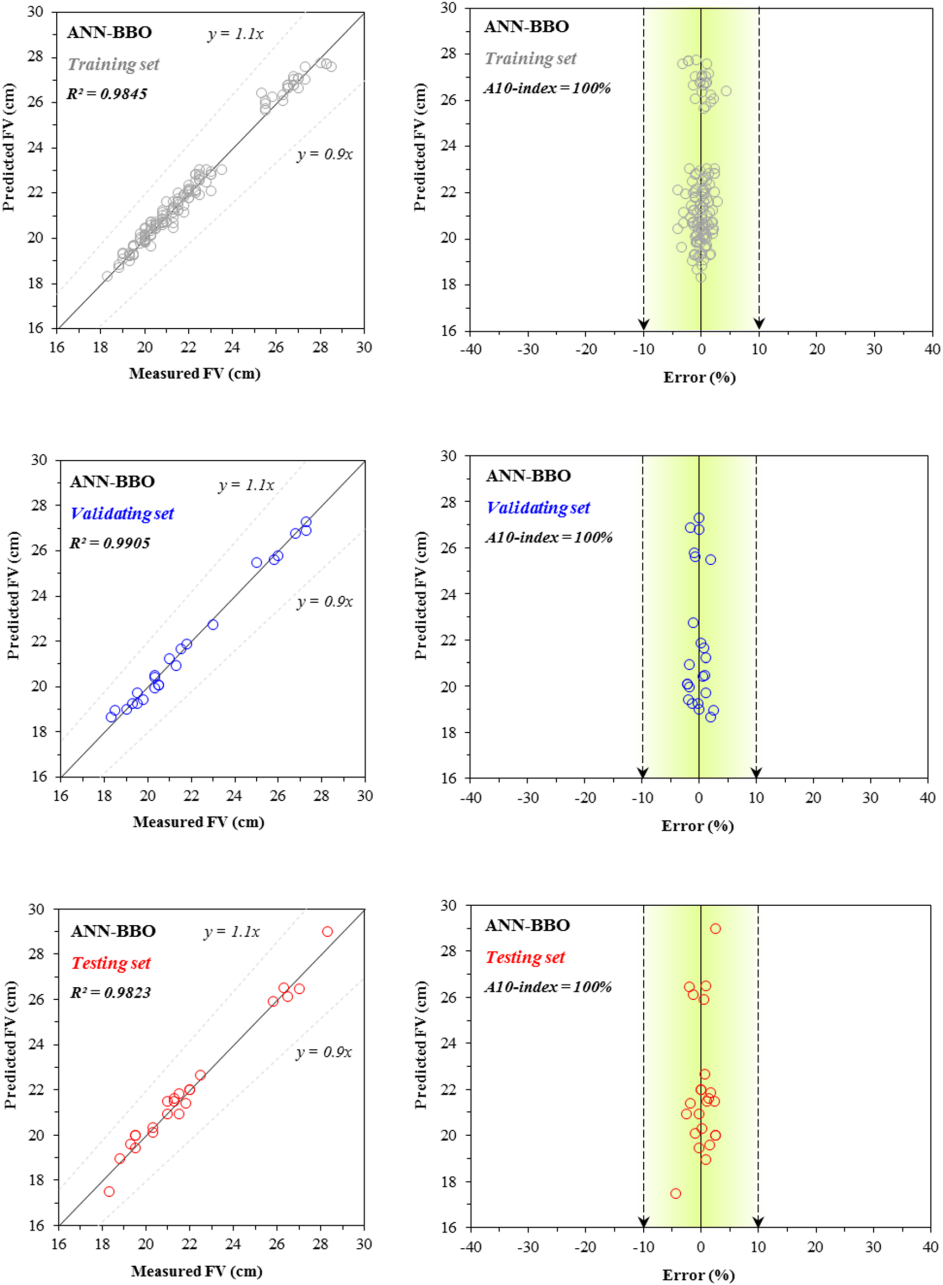
Comparison between the measured and predicted ANN–BBO values for FV, including the error distribution.

**Fig. 12:**
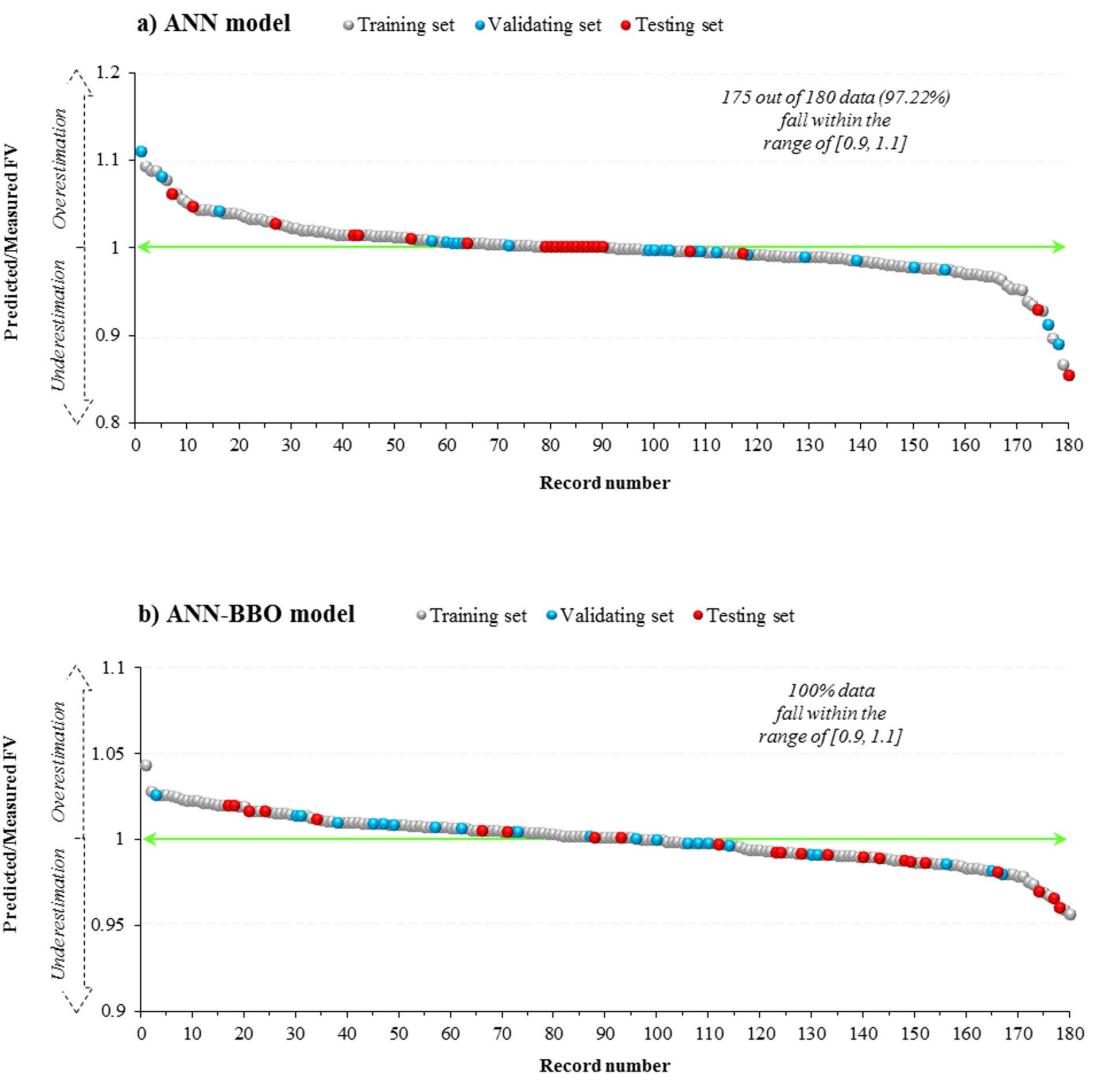
Ratios of predicted/measured FV value for the models (**a**) ANN and (**b**) ANN–BBO.

A similar analysis is performed for the FV property of cementitious systems incorporating fly ash in Figs. 10, 11, 12. The correlation values for the single ANN model’s performance are 0.9219 for the training set, 0.91 for the validating set, and 0.9183 for the testing set (Fig. 10), while the corresponding values for the hybrid ANN–BBO model are 0.9845, 0.9905, and 0.9823, respectively (Fig. 11). A comparison of the two figures shows that the hybrid model yields a higher concentration of data records within the region bounded by the black dotted lines—representing a ± 10% deviation from the *y* = *x* line—indicating that the ANN–BBO model outperforms the single ANN model in estimating the FV of cementitious systems containing fly ash. This is further evident in the error distribution graphs, which show that the predictions from the single model exhibit a wider spread (indicating higher error) compared to those from the hybrid model.

Figure 12 illustrates that the predicted to measured FV value ratio varies roughly between 0.85 and 1.1 for the single ANN model, while it falls within a narrower range of approximately 0.95 to 1.05 for the hybrid ANN–BBO model. The ratio values in the hybrid model are noticeably closer to the ideal line of *y* = 1, indicating strong alignment of the predicted to the measured values. The results of FV performance modeling are consistent with earlier results regarding the effectiveness of the ANN–BBO model in predicting the CS of cementitious systems incorporating fly ash.

To gain deeper insight into the performance of the proposed models, several evaluation criteria were applied, with the results summarized in Table 7. Grasping the ideal value of these criteria enables more effective evaluation. Specifically, the best performance for MAE and RMSE is indicated by values nearing zero, while ideal values for NSE are close to 1, and for VAF and the A10-index, they should approach 100%. By comparing the evaluation criteria across all sets, it is evident that the hybrid proposed model has considerably reduced the prediction errors and consequently achieved higher accuracy than the classical ANN model. For example, the results emphasize that the ANN–BBO model outperformed the standalone ANN model, demonstrating approximately a 67% reduction in both MAE (dropping from 0.1486 in ANN to 0.0490 in ANN–BBO) and RMSE (from 4.40 in ANN to 1.36 in ANN–BBO) across all CS data. Likewise, the FV modeling results exhibit a decrease of around 50%. In addition, as presented in Table 7, the hybrid ANN–BBO model consistently outperformed the standalone ANN across all datasets for both output variables. For CS prediction, the ANN–BBO achieved remarkably higher R^2^ (0.992 [95% CI 0.988–0.995] for all data) and substantially lower RMSE values (1.36 [95% CI 1.11–1.66]), indicating superior accuracy and generalization capability. Similarly, for FV prediction, the ANN–BBO model maintained excellent agreement with experimental data (R^2^ = 0.985 [95% CI 0.981–0.989]; RMSE = 0.32 [95% CI 0.28–0.36]), surpassing the standalone ANN by a significant margin. The narrow 95% confidence intervals confirm the statistical robustness, reliability, and stability of the proposed ANN–BBO model. Therefore, the evaluation metrics provide additional support for the earlier findings and reinforce the effectiveness of the proposed hybrid model in predicting the properties of cementitious systems containing fly ash.

**Table 7:** Comparing the evaluation criteria.

Output	Proposed model	Data set	Metrics				95% confidence intervals [95% CI]	
			MAE*	NSE	VAF (%)	A10-index (%)	R^2^ [95% CI]	RMSE [95% CI]
*Y*_1_: Compressive strength	ANN	Train	0.1473	0.9171	91.9012	44.78	0.933[0.909, 0.955]	4.36[3.58, 5.08]
		Validate	0.1000	0.8547	86.4957	65.22	0.905[0.829, 0.959]	3.89[2.64, 5.06]
		Test	0.1850	0.9010	91.1665	26.08	0.918[0.844, 0.960]	4.47[3.51, 5.32]
		All	0.1486	0.9123	91.6403	44.44	0.930[0.909, 0.950]	4.40[3.74, 5.00]
	ANN–BBO	Train	0.0522	0.9915	99.1561	85.07	0.992[0.987, 0.995]	1.38[1.06, 1.73]
		Validate	0.0277	0.9895	98.9570	95.65	0.990[0.979, 0.996]	1.02[0.68, 1.35]
		Test	0.0501	0.9902	99.0193	86.96	0.991[0.981, 0.997]	1.44[0.81, 2.04]
		All	0.0490	0.9915	99.1548	86.67	0.992[0.988, 0.995]	1.36[1.11, 1.66]
*Y*_2_: Flow value	ANN	Train	0.0226	0.9214	92.1574	98.50	0.921[0.888, 0.949]	0.71[0.59, 0.83]
		Validate	0.0249	0.9092	91.0000	91.30	0.911[0.794, 0.975]	0.85[0.43, 1.21]
		Test	0.0151	0.9113	91.1644	95.65	0.918[0.741, 0.991]	0.78[0.32, 1.32]
		All	0.0223	0.9180	91.8287	97.22	0.919[0.888, 0.919]	0.74[0.62, 0.86]
	ANN–BBO	Train	0.0107	0.9844	98.4403	100.00	0.985[0.981, 0.990]	0.31[0.26, 0.35]
		Validate	0.0115	0.9902	99.0445	100.00	0.991[0.982, 0.995]	0.29[0.23, 0.34]
		Test	0.0110	0.9817	98.1931	100.00	0.982[0.963, 0.992]	0.37[0.28, 0.47]
		All	0.0112	0.9849	98.4930	100.00	0.985[0.981, 0.989]	0.32[0.28, 0.36]

*The unit of MAE and RMSE metrics is megapascal (MPa) for compressive strength and centimeter (cm) for flow value.

### Contribution of input variables in modeling the CS and FV of cementitious systems containing fly ash

**Fig. 13:**
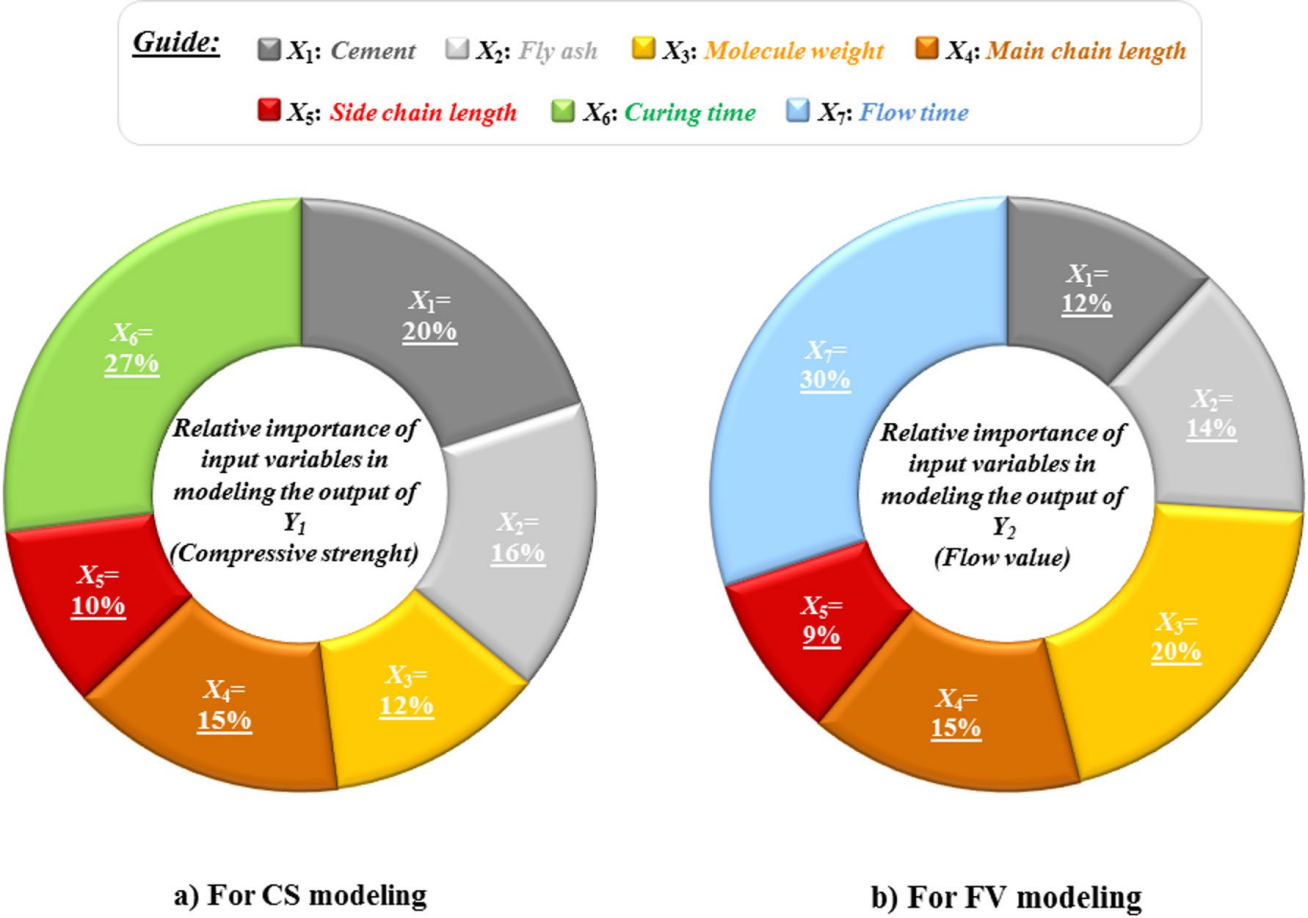
Importance of input variables in modeling the (**a**) CS and (**b**) FV.

To evaluate the contribution of input variables to the performance of models predicting the CS and FV of cementitious systems containing fly ash, a comparative analysis was performed. Accordingly, the impact of each input on the best model’s performance is assessed by evaluating how the model behaves when that specific input is excluded from the modeling process. This method helps identify variables whose removal significantly reduces the model’s performance, indicating their important role in the prediction process. Figure 13 presents the importance of the variables considered in the most effective model (ANN–BBO). While all input variables play a role in the model’s performance, their levels of influence differ. As shown in Fig. 13, curing time (*X*_6_) and flow time (*X*_7_) are the most influential variables affecting CS and FV, contributing 27% and 30%, respectively. Next in importance for predicting CS are cement (*X*_1_ = 20%) and fly ash (*X*_2_ = 16%) as cementitious materials. Similarly, molecular weight (*X*_3_ = 20%) and main chain length (*X*_4_ = 15%) are key characteristics of HRWRAs influencing the prediction of FV. Altogether, the three input variables—curing time, cement, and fly ash—for CS, and flow time, molecular weight, and main chain length for FV, contribute approximately 65% to the predictive accuracy for estimating CS and FV in cementitious systems containing fly ash. The remaining variables have a comparatively lower impact on the predictions.

**Fig. 14:**
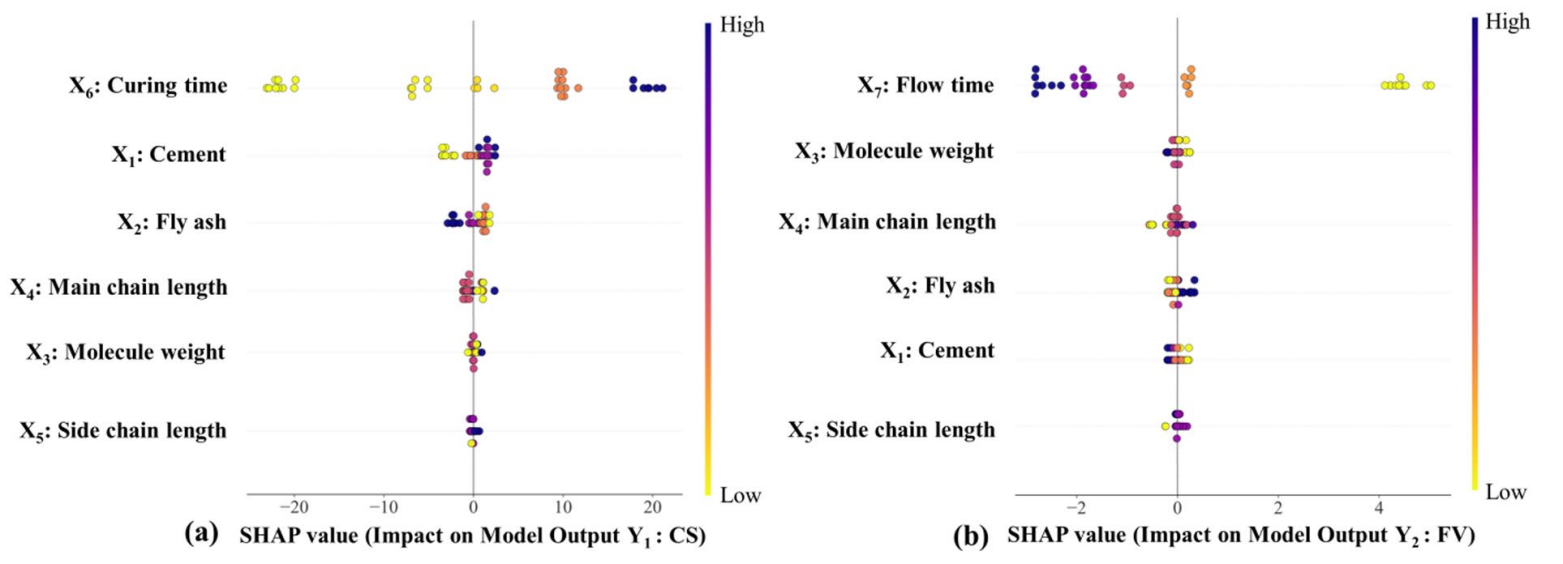
SHAP summary plot for (**a**) CS and (**b**) FV.

To gain deeper insights into the contribution of individual input parameters, the SHapley Additive exPlanations (SHAP) technique—derived from cooperative game theory—was applied to quantify and interpret each variable’s influence on the model output^63^. Figure 14 visualizes the relative importance of all features by depicting their respective impacts on the prediction outcomes. The color scale represents the magnitude of each feature, transitioning from low (yellow) to high (dark purple) values. The horizontal axis indicates the directional effect of each variable on the model prediction, where positive and negative contributions correspond to increases or decreases in the predicted response, respectively. For CS, the curing time (*X*_6_) emerged as the most influential parameter, with the dominance of dark-purple points on the positive side confirming its enhancing effect on strength development. The subsequent key contributors were cement content (*X*_1_) and fly ash (*X*_2_), both of which are primary constituents governing the mechanical performance of cementitious systems. Conversely, for FV, the most critical variable was the flow time (*X*_7_), exhibiting a predominant negative influence. The following influential parameters for FV were the HRWRA-related features—molecular weight (*X*_3_) and main chain length (*X*_4_)—which strongly affect the rheological behavior of the mixtures. These findings collectively demonstrate that the SHAP analysis provides a consistent and interpretable framework for identifying the dominant factors governing both mechanical and rheological properties in fly ash–based cementitious composites.

**Fig. 15:**
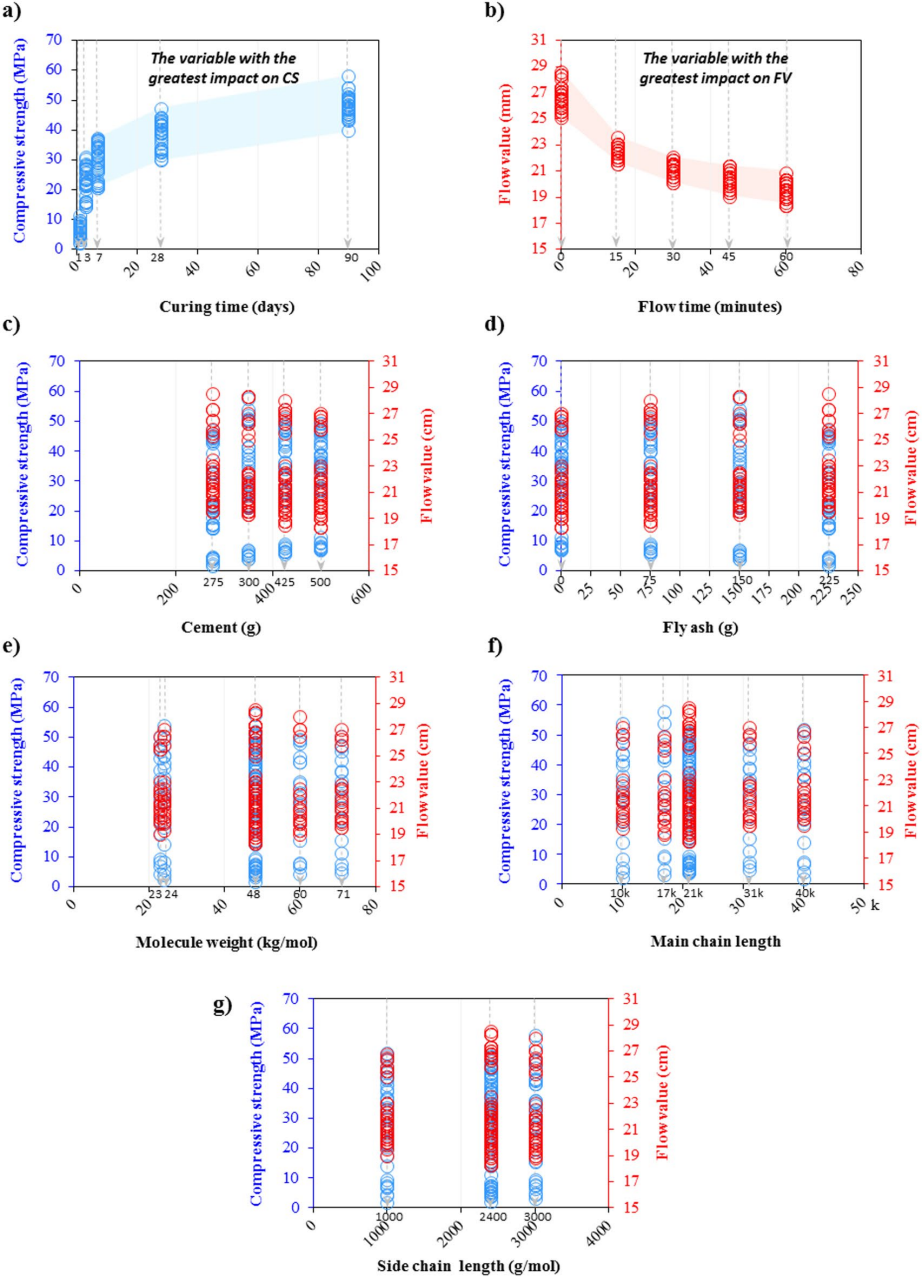
Effect of variables used in modeling on CS and FV outputs.

In this regard, Fig. 15 enables a more expressive analysis of the effect of the variables on the CS and FV outputs. As can be seen in Fig. 15a,b, CS exhibits the highest sensitivity to changes in curing time, while FV is most sensitive to variations in flow time. The main difference lies in the fact that the CS of cementitious systems increases with longer curing time. On the other hand, flow time is inversely related to FV, meaning that greater flow times reflect lower flowability. For example, the most notable changes in CS take place in the early stages of curing, especially from day 1 to day 7, whereas the most pronounced variations in FV are observed within the first 30 min. Mixtures containing 425 g of cement and 150 g of fly ash demonstrated the highest CS. Following the flow time variable, the FV is influenced by two factors of molecular weight and main chain length. The analyses presented in this section can be valuable because they clarify the impact of each variable used on the model’s output, providing clearer insight into the role of these variables and how they affect the model’s predictions.

## Conclusions

The compressive strength and flow value of cementitious systems were statistically modeled using data collected from the experimental investigation of the effects of fly ash substitution and the chemical structure of HRWRAs.

In conclusion, the ANN model achieved R^2^ values of 0.9325, 0.9048, and 0.9180 for CS during training, validation, and testing phases, respectively. For FV, the ANN model predicted R^2^ values of 0.9219, 0.9100, and 0.9183 at training, validation, and testing stages, respectively. In contrast, the ANN–BBO hybrid model substantially outperformed the ANN alone, attaining R^2^ values of 0.9917, 0.9897, and 0.9903 for CS across training, validation, and testing sets, respectively. For FV, the ANN–BBO model yielded R^2^ values of 0.9845, 0.9905, and 0.9823 at the corresponding phases, indicating an almost perfect correlation with the ideal regression line (y = x). Error distribution analysis further confirmed that over 85% of ANN–BBO predictions for CS and 100% for FV fell within narrow deviation intervals of [-10%, 10%], demonstrating the hybrid model’s performance accuracy and reliability.

Analysis of performance metrics across all data subsets revealed significant enhancements in prediction accuracy and consistency when using the ANN–BBO approach. Specifically, metrics including mean absolute error (MAE), root mean square error (RMSE), Nash–Sutcliffe efficiency (NSE), variance accounted for (VAF), and A10-index confirmed reductions in error magnitude and improvements in model robustness, with ANN–BBO reducing prediction errors by more than 60% in certain metrics compared to the standalone ANN. These results validate the efficacy of integrating metaheuristic optimization with neural network models, particularly for complex cementitious systems involving multiple interacting variables.

The ANN–BBO hybrid methodology offers a more precise, stable, and generalizable predictive framework for modeling the performance of fly ash-based cementitious mortars containing HRWRAs with varied molecular structures. This modeling technique facilitates the development of sustainable, high-performance construction materials and serves as a powerful tool for optimizing admixture design within modern concrete technology.

This study demonstrates that ANN–BBO surrogates can reliably predict compressive strength and flow of FA-incorporated mortars while explicitly accounting for the molecular architecture of PCE-type HRWRAs. In practice, these models enable producers to meet target performance with lower binder demand and/or higher FA replacement, thereby reducing clinker intensity and embodied CO_2_. Because RMSE directly translates into reliability-based safety margins, the observed error reductions versus a baseline ANN indicate a smaller overdesign requirement, which helps curb unnecessary cement additions during mix qualification and production. The workflow is straightforward to integrate into ready-mix and precast QC, supporting data-driven proportioning, fewer re-tests/re-tempering events, and more consistent field performance—all aligned with sustainable concrete production.

## Future works

Our ANN–BBO surrogates accurately predict compressive strength and flow of FA-incorporated mortars while encoding HRWRA molecular architecture, enabling target performance with less cement or higher FA, thereby reducing clinker intensity and embodied CO_2_. Because RMSE reductions map directly to smaller reliability-based safety margins, the hybrid model curtails overdesign and unnecessary binder additions in production. Going forward, we will broaden the dataset across cements (CEM I/II, LC^3^), wider FA ranges, additional HRWRA families, and multi-lab sources to assess domain shift. We will benchmark alternative learners (gradient-boosted trees, GPR, transformer-style models) with Bayesian/evolutionary hyperparameter search, ensembles, and multi-objective optimization of strength–flow–CO_2_–cost. Finally, we will quantify uncertainty and calibration (e.g., conformal intervals), couple the surrogate with LCA/LCCA, and conduct field trials with active-learning–guided experiments to accelerate deployment.

While the present dataset centers on Class F FA, the proposed ANN/ANN–BBO framework is SCM-agnostic provided the inputs include descriptors that capture the SCM’s physicochemical action. As FA supply tightens, the model can be extended to blends and FA-lean/FA-free systems (e.g., slag, metakaolin/calcined clay, limestone, ground glass pozzolan) by replacing X_2_ (Fly ash) with a feature vector of SCM properties (e.g., oxide composition, glass content, fineness/PSD, LOI, specific surface, morphology). We outline a practical transfer-learning route: (i) pretrain on the FA corpus, (ii) add new SCM data, (iii) fine-tune with domain-adaptation regularization and monitor drift. For deployment, we recommend domain-shift checks (error/feature monitors) and light local recalibration when the SCM set changes. The surrogate also remains useful for legacy FA concretes and reclaimed/beneficiated ashes where they are available. In future work we will build a multi-SCM surrogate with SCM embeddings and active-learning to target under-sampled regions of the mix space, enabling performance–carbon optimization regardless of the specific SCM portfolio.

## Supplementary Information

Below is the link to the electronic supplementary material.

Supplementary Material 1

## Data Availability

The database used in this study is available in the supplementary file.

## Abbreviations

WRA: Water-reducing admixture
HWRA: High-range water-reducing admixture
CS: Compressive strength
AI: Artificial intelligence
ANN: Artificial neural network
LR: Linear regression
NLR: Nonlinear regression
RFR: Random forest regression
ETR: Extra trees regression
GBR: Gradient boosting regression
XGB: EXtreme gradient boosting
XGBR: EXtreme gradient boosting regression
LSSVM: Least square support vector machine
CSA: Coupled simulated annealing
MPMR: Minimax probability machine regression
RVM: Relevance vector machine
ENN: Emotional neural network
GP: Genetic programming
MLR: Multi-linear regression
PSO: Particle swarm optimization
ANFIS: Adaptive neuro-fuzzy inference system
GA: Genetic algorithm
DT: Decision tree
SVM: Support vector machines
MLP: Multi-layer perceptron
BP: Backpropagation
FQ: Full-quadratic
IN: Interaction
KNN: K-nearest neighbors
BBO: Biogeography-based optimization
CNN: Convolutional neural networks
ELM: Extreme learning machine
GEP: Gene expression programming
M5p: M5prime
MAE: Mean absolute error
NSE: Nash–Sutcliffe efficiency
RF: Random forest
RMSE: Root mean squared error
SIVs: Suitability index variables
SVR: Support vector machines
VAF: Variance accounted for
