# Supplementary material for: ANN trained by BBO for modeling of fly ash cementitious systems with high range water reducing admixtures
Source: Sci Rep. 2026 Feb 1;16:4540. doi: 10.1038/s41598-025-32972-1 (PMC12868753; doi:10.1038/s41598-025-32972-1)
Supplement: Supplementary file 1 — Supplementary Material 1 [file 41598_2025_32972_MOESM1_ESM.docx]

**Table S1.** The experimental data were used in the study

|  | **Cement** | **Fly ash** | **Additive properties** | | |  |  |  |  |
| --- | --- | --- | --- | --- | --- | --- | --- | --- | --- |
| **Mixture** | **Dosage (g)** | **Dosage (g)** | **Molecule weight (kg/mol)** | **Main chain length** | **Side chain length (g/mol)** | **Curing time (days)** | **Flow time (seconds)** | **Compressive strength (MPa)** | **Flow value (cm)** |
| **1** | **500** | **0** | **24** | **10k** | **2400** | **1** | **0** | **8,3** | **25,8** |
| **2** | **500** | **0** | **24** | **10k** | **2400** | **3** | **15** | **30,1** | **21,8** |
| **3** | **500** | **0** | **24** | **10k** | **2400** | **7** | **30** | **36,4** | **21,3** |
| **4** | **500** | **0** | **24** | **10k** | **2400** | **28** | **45** | **41,2** | **20,0** |
| **5** | **500** | **0** | **24** | **10k** | **2400** | **90** | **60** | **47,2** | **19,3** |
| **6** | **500** | **0** | **48** | **21k** | **2400** | **1** | **0** | **7,0** | **26,8** |
| **7** | **500** | **0** | **48** | **21k** | **2400** | **3** | **15** | **27,8** | **22,5** |
| **8** | **500** | **0** | **48** | **21k** | **2400** | **7** | **30** | **33,7** | **20,5** |
| **9** | **500** | **0** | **48** | **21k** | **2400** | **28** | **45** | **38,8** | **19,5** |
| **10** | **500** | **0** | **48** | **21k** | **2400** | **90** | **60** | **46,5** | **18,3** |
| **11** | **500** | **0** | **71** | **31k** | **2400** | **1** | **0** | **11,2** | **26,5** |
| **12** | **500** | **0** | **71** | **31k** | **2400** | **3** | **15** | **30,5** | **22,8** |
| **13** | **500** | **0** | **71** | **31k** | **2400** | **7** | **30** | **35,4** | **21,8** |
| **14** | **500** | **0** | **71** | **31k** | **2400** | **28** | **45** | **38,5** | **20,3** |
| **15** | **500** | **0** | **71** | **31k** | **2400** | **90** | **60** | **45,2** | **19,8** |
| **16** | **425** | **75** | **24** | **10k** | **2400** | **1** | **0** | **5,6** | **27,0** |
| **17** | **425** | **75** | **24** | **10k** | **2400** | **3** | **15** | **28,1** | **23,0** |
| **18** | **425** | **75** | **24** | **10k** | **2400** | **7** | **30** | **33,9** | **21,5** |
| **19** | **425** | **75** | **24** | **10k** | **2400** | **28** | **45** | **43,9** | **21,3** |
| **20** | **425** | **75** | **24** | **10k** | **2400** | **90** | **60** | **49,3** | **20,3** |
| **21** | **425** | **75** | **48** | **21k** | **2400** | **1** | **0** | **6,4** | **26,8** |
| **22** | **425** | **75** | **48** | **21k** | **2400** | **3** | **15** | **23,0** | **22,0** |
| **23** | **425** | **75** | **48** | **21k** | **2400** | **7** | **30** | **31,0** | **20,8** |
| **24** | **425** | **75** | **48** | **21k** | **2400** | **28** | **45** | **40,0** | **19,5** |
| **25** | **425** | **75** | **48** | **21k** | **2400** | **90** | **60** | **47,2** | **18,5** |
| **26** | **425** | **75** | **71** | **31k** | **2400** | **1** | **0** | **7,5** | **27,0** |
| **27** | **425** | **75** | **71** | **31k** | **2400** | **3** | **15** | **26,3** | **22,5** |
| **28** | **425** | **75** | **71** | **31k** | **2400** | **7** | **30** | **33,9** | **21,5** |
| **29** | **425** | **75** | **71** | **31k** | **2400** | **28** | **45** | **42,2** | **20,8** |
| **30** | **425** | **75** | **71** | **31k** | **2400** | **90** | **60** | **47,0** | **20,3** |
| **31** | **350** | **150** | **24** | **10k** | **2400** | **1** | **0** | **4,0** | **26,5** |
| **32** | **350** | **150** | **24** | **10k** | **2400** | **3** | **15** | **20,0** | **22,0** |
| **33** | **350** | **150** | **24** | **10k** | **2400** | **7** | **30** | **28,8** | **21,3** |
| **34** | **350** | **150** | **24** | **10k** | **2400** | **28** | **45** | **39,8** | **20,5** |
| **35** | **350** | **150** | **24** | **10k** | **2400** | **90** | **60** | **53,8** | **19,8** |
| **36** | **350** | **150** | **48** | **21k** | **2400** | **1** | **0** | **5,2** | **28,3** |
| **37** | **350** | **150** | **48** | **21k** | **2400** | **3** | **15** | **21,1** | **22,3** |
| **38** | **350** | **150** | **48** | **21k** | **2400** | **7** | **30** | **26,5** | **21,3** |
| **39** | **350** | **150** | **48** | **21k** | **2400** | **28** | **45** | **37,5** | **20,3** |
| **40** | **350** | **150** | **48** | **21k** | **2400** | **90** | **60** | **50,5** | **19,3** |
| **41** | **350** | **150** | **71** | **31k** | **2400** | **1** | **0** | **6,0** | **26,3** |
| **42** | **350** | **150** | **71** | **31k** | **2400** | **3** | **15** | **22,2** | **22,3** |
| **43** | **350** | **150** | **71** | **31k** | **2400** | **7** | **30** | **29,1** | **21,0** |
| **44** | **350** | **150** | **71** | **31k** | **2400** | **28** | **45** | **38,8** | **20,0** |
| **45** | **350** | **150** | **71** | **31k** | **2400** | **90** | **60** | **50,2** | **19,5** |
| **46** | **275** | **225** | **24** | **10k** | **2400** | **1** | **0** | **2,2** | **26,5** |
| **47** | **275** | **225** | **24** | **10k** | **2400** | **3** | **15** | **14,1** | **22,5** |
| **48** | **275** | **225** | **24** | **10k** | **2400** | **7** | **30** | **21,5** | **21,5** |
| **49** | **275** | **225** | **24** | **10k** | **2400** | **28** | **45** | **31,4** | **20,8** |
| **50** | **275** | **225** | **24** | **10k** | **2400** | **90** | **60** | **43,7** | **20,3** |
| **51** | **275** | **225** | **48** | **21k** | **2400** | **1** | **0** | **4,2** | **28,5** |
| **52** | **275** | **225** | **48** | **21k** | **2400** | **3** | **15** | **14,7** | **23,5** |
| **53** | **275** | **225** | **48** | **21k** | **2400** | **7** | **30** | **20,4** | **21,5** |
| **54** | **275** | **225** | **48** | **21k** | **2400** | **28** | **45** | **33,2** | **21,3** |
| **55** | **275** | **225** | **48** | **21k** | **2400** | **90** | **60** | **44,2** | **20,3** |
| **56** | **275** | **225** | **71** | **31k** | **2400** | **1** | **0** | **4,7** | **25,8** |
| **57** | **275** | **225** | **71** | **31k** | **2400** | **3** | **15** | **15,5** | **22,5** |
| **58** | **275** | **225** | **71** | **31k** | **2400** | **7** | **30** | **22,7** | **21,3** |
| **59** | **275** | **225** | **71** | **31k** | **2400** | **28** | **45** | **33,2** | **20,3** |
| **60** | **275** | **225** | **71** | **31k** | **2400** | **90** | **60** | **46,9** | **19,5** |
| **61** | **500** | **0** | **23** | **21k** | **1000** | **1** | **0** | **9,3** | **25,8** |
| **62** | **500** | **0** | **23** | **21k** | **1000** | **3** | **15** | **27,7** | **22,0** |
| **63** | **500** | **0** | **23** | **21k** | **1000** | **7** | **30** | **36,2** | **21,0** |
| **64** | **500** | **0** | **23** | **21k** | **1000** | **28** | **45** | **38,9** | **20,0** |
| **65** | **500** | **0** | **23** | **21k** | **1000** | **90** | **60** | **46,0** | **19,0** |
| **66** | **500** | **0** | **48** | **21k** | **2400** | **1** | **0** | **7,3** | **26,8** |
| **67** | **500** | **0** | **48** | **21k** | **2400** | **3** | **15** | **27,6** | **21,8** |
| **68** | **500** | **0** | **48** | **21k** | **2400** | **7** | **30** | **34,7** | **20,0** |
| **69** | **500** | **0** | **48** | **21k** | **2400** | **28** | **45** | **38,7** | **19,0** |
| **70** | **500** | **0** | **48** | **21k** | **2400** | **90** | **60** | **46,8** | **18,3** |
| **71** | **500** | **0** | **60** | **21k** | **3000** | **1** | **0** | **7,9** | **27,0** |
| **72** | **500** | **0** | **60** | **21k** | **3000** | **3** | **15** | **27,1** | **22,0** |
| **73** | **500** | **0** | **60** | **21k** | **3000** | **7** | **30** | **34,3** | **20,5** |
| **74** | **500** | **0** | **60** | **21k** | **3000** | **28** | **45** | **41,6** | **19,8** |
| **75** | **500** | **0** | **60** | **21k** | **3000** | **90** | **60** | **48,1** | **19,0** |
| **76** | **425** | **75** | **23** | **21k** | **1000** | **1** | **0** | **8,3** | **26,5** |
| **77** | **425** | **75** | **23** | **21k** | **1000** | **3** | **15** | **25,4** | **22,3** |
| **78** | **425** | **75** | **23** | **21k** | **1000** | **7** | **30** | **30,9** | **21,3** |
| **79** | **425** | **75** | **23** | **21k** | **1000** | **28** | **45** | **43,0** | **20,3** |
| **80** | **425** | **75** | **23** | **21k** | **1000** | **90** | **60** | **47,2** | **19,8** |
| **81** | **425** | **75** | **48** | **21k** | **2400** | **1** | **0** | **6,4** | **27,3** |
| **82** | **425** | **75** | **48** | **21k** | **2400** | **3** | **15** | **23,5** | **22,0** |
| **83** | **425** | **75** | **48** | **21k** | **2400** | **7** | **30** | **31,2** | **20,5** |
| **84** | **425** | **75** | **48** | **21k** | **2400** | **28** | **45** | **40,0** | **19,3** |
| **85** | **425** | **75** | **48** | **21k** | **2400** | **90** | **60** | **47,9** | **18,8** |
| **86** | **425** | **75** | **60** | **21k** | **3000** | **1** | **0** | **7,6** | **28,0** |
| **87** | **425** | **75** | **60** | **21k** | **3000** | **3** | **15** | **21,9** | **22,5** |
| **88** | **425** | **75** | **60** | **21k** | **3000** | **7** | **30** | **31,3** | **21,0** |
| **89** | **425** | **75** | **60** | **21k** | **3000** | **28** | **45** | **41,7** | **19,8** |
| **90** | **425** | **75** | **60** | **21k** | **3000** | **90** | **60** | **49,1** | **19,3** |
| **91** | **350** | **150** | **23** | **21k** | **1000** | **1** | **0** | **7,0** | **25,5** |
| **92** | **350** | **150** | **23** | **21k** | **1000** | **3** | **15** | **21,4** | **22,5** |
| **93** | **350** | **150** | **23** | **21k** | **1000** | **7** | **30** | **27,5** | **21,5** |
| **94** | **350** | **150** | **23** | **21k** | **1000** | **28** | **45** | **33,5** | **20,5** |
| **95** | **350** | **150** | **23** | **21k** | **1000** | **90** | **60** | **50,0** | **20,3** |
| **96** | **350** | **150** | **48** | **21k** | **2400** | **1** | **0** | **5,4** | **28,3** |
| **97** | **350** | **150** | **48** | **21k** | **2400** | **3** | **15** | **21,2** | **22,3** |
| **98** | **350** | **150** | **48** | **21k** | **2400** | **7** | **30** | **26,8** | **21,5** |
| **99** | **350** | **150** | **48** | **21k** | **2400** | **28** | **45** | **36,5** | **20,0** |
| **100** | **350** | **150** | **48** | **21k** | **2400** | **90** | **60** | **50,4** | **19,5** |
| **101** | **350** | **150** | **60** | **21k** | **3000** | **1** | **0** | **6,5** | **27,0** |
| **102** | **350** | **150** | **60** | **21k** | **3000** | **3** | **15** | **22,2** | **21,5** |
| **103** | **350** | **150** | **60** | **21k** | **3000** | **7** | **30** | **27,9** | **21,0** |
| **104** | **350** | **150** | **60** | **21k** | **3000** | **28** | **45** | **35,2** | **20,0** |
| **105** | **350** | **150** | **60** | **21k** | **3000** | **90** | **60** | **49,2** | **19,3** |
| **106** | **275** | **225** | **23** | **21k** | **1000** | **1** | **0** | **4,4** | **25,5** |
| **107** | **275** | **225** | **23** | **21k** | **1000** | **3** | **15** | **17,7** | **23,0** |
| **108** | **275** | **225** | **23** | **21k** | **1000** | **7** | **30** | **22,0** | **22,0** |
| **109** | **275** | **225** | **23** | **21k** | **1000** | **28** | **45** | **29,9** | **21,3** |
| **110** | **275** | **225** | **23** | **21k** | **1000** | **90** | **60** | **45,2** | **20,8** |
| **111** | **275** | **225** | **48** | **21k** | **2400** | **1** | **0** | **4,1** | **27,3** |
| **112** | **275** | **225** | **48** | **21k** | **2400** | **3** | **15** | **15,6** | **22,8** |
| **113** | **275** | **225** | **48** | **21k** | **2400** | **7** | **30** | **21,0** | **21,0** |
| **114** | **275** | **225** | **48** | **21k** | **2400** | **28** | **45** | **32,5** | **20,0** |
| **115** | **275** | **225** | **48** | **21k** | **2400** | **90** | **60** | **44,8** | **19,5** |
| **116** | **275** | **225** | **60** | **21k** | **3000** | **1** | **0** | **4,4** | **26,5** |
| **117** | **275** | **225** | **60** | **21k** | **3000** | **3** | **15** | **15,6** | **22,0** |
| **118** | **275** | **225** | **60** | **21k** | **3000** | **7** | **30** | **21,2** | **20,8** |
| **119** | **275** | **225** | **60** | **21k** | **3000** | **28** | **45** | **29,7** | **20,3** |
| **120** | **275** | **225** | **60** | **21k** | **3000** | **90** | **60** | **43,4** | **20,0** |
| **121** | **500** | **0** | **48** | **40k** | **1000** | **1** | **0** | **7,5** | **26,8** |
| **122** | **500** | **0** | **48** | **40k** | **1000** | **3** | **15** | **26,8** | **23,0** |
| **123** | **500** | **0** | **48** | **40k** | **1000** | **7** | **30** | **36,9** | **21,5** |
| **124** | **500** | **0** | **48** | **40k** | **1000** | **28** | **45** | **43,6** | **20,8** |
| **125** | **500** | **0** | **48** | **40k** | **1000** | **90** | **60** | **49,4** | **20,0** |
| **126** | **500** | **0** | **48** | **21k** | **2400** | **1** | **0** | **7,2** | **26,8** |
| **127** | **500** | **0** | **48** | **21k** | **2400** | **3** | **15** | **26,8** | **21,8** |
| **128** | **500** | **0** | **48** | **21k** | **2400** | **7** | **30** | **33,7** | **20,3** |
| **129** | **500** | **0** | **48** | **21k** | **2400** | **28** | **45** | **38,8** | **19,3** |
| **130** | **500** | **0** | **48** | **21k** | **2400** | **90** | **60** | **46,7** | **18,3** |
| **131** | **500** | **0** | **48** | **17k** | **3000** | **1** | **0** | **9,5** | **26,0** |
| **132** | **500** | **0** | **48** | **17k** | **3000** | **3** | **15** | **28,3** | **22,0** |
| **133** | **500** | **0** | **48** | **17k** | **3000** | **7** | **30** | **36,1** | **20,8** |
| **134** | **500** | **0** | **48** | **17k** | **3000** | **28** | **45** | **42,7** | **20,0** |
| **135** | **500** | **0** | **48** | **17k** | **3000** | **90** | **60** | **49,2** | **19,0** |
| **136** | **425** | **75** | **48** | **40k** | **1000** | **1** | **0** | **6,7** | **26,3** |
| **137** | **425** | **75** | **48** | **40k** | **1000** | **3** | **15** | **25,7** | **22,0** |
| **138** | **425** | **75** | **48** | **40k** | **1000** | **7** | **30** | **36,5** | **21,3** |
| **139** | **425** | **75** | **48** | **40k** | **1000** | **28** | **45** | **43,9** | **20,5** |
| **140** | **425** | **75** | **48** | **40k** | **1000** | **90** | **60** | **51,0** | **20,0** |
| **141** | **425** | **75** | **48** | **21k** | **2400** | **1** | **0** | **6,4** | **27,3** |
| **142** | **425** | **75** | **48** | **21k** | **2400** | **3** | **15** | **23,8** | **22,3** |
| **143** | **425** | **75** | **48** | **21k** | **2400** | **7** | **30** | **32,2** | **20,5** |
| **144** | **425** | **75** | **48** | **21k** | **2400** | **28** | **45** | **40,1** | **19,5** |
| **145** | **425** | **75** | **48** | **21k** | **2400** | **90** | **60** | **48,2** | **18,8** |
| **146** | **425** | **75** | **48** | **17k** | **3000** | **1** | **0** | **8,8** | **25,5** |
| **147** | **425** | **75** | **48** | **17k** | **3000** | **3** | **15** | **26,6** | **21,5** |
| **148** | **425** | **75** | **48** | **17k** | **3000** | **7** | **30** | **35,9** | **20,0** |
| **149** | **425** | **75** | **48** | **17k** | **3000** | **28** | **45** | **46,8** | **19,5** |
| **150** | **425** | **75** | **48** | **17k** | **3000** | **90** | **60** | **53,8** | **18,8** |
| **151** | **350** | **150** | **48** | **40k** | **1000** | **1** | **0** | **4,1** | **25,0** |
| **152** | **350** | **150** | **48** | **40k** | **1000** | **3** | **15** | **21,7** | **22,3** |
| **153** | **350** | **150** | **48** | **40k** | **1000** | **7** | **30** | **25,6** | **21,5** |
| **154** | **350** | **150** | **48** | **40k** | **1000** | **28** | **45** | **41,2** | **20,5** |
| **155** | **350** | **150** | **48** | **40k** | **1000** | **90** | **60** | **52,0** | **19,5** |
| **156** | **350** | **150** | **48** | **21k** | **2400** | **1** | **0** | **5,1** | **28,3** |
| **157** | **350** | **150** | **48** | **21k** | **2400** | **3** | **15** | **21,4** | **22,5** |
| **158** | **350** | **150** | **48** | **21k** | **2400** | **7** | **30** | **26,7** | **21,3** |
| **159** | **350** | **150** | **48** | **21k** | **2400** | **28** | **45** | **41,4** | **20,3** |
| **160** | **350** | **150** | **48** | **21k** | **2400** | **90** | **60** | **50,8** | **19,5** |
| **161** | **350** | **150** | **48** | **17k** | **3000** | **1** | **0** | **4,8** | **26,3** |
| **162** | **350** | **150** | **48** | **17k** | **3000** | **3** | **15** | **23,2** | **21,8** |
| **163** | **350** | **150** | **48** | **17k** | **3000** | **7** | **30** | **29,7** | **21,0** |
| **164** | **350** | **150** | **48** | **17k** | **3000** | **28** | **45** | **41,5** | **20,0** |
| **165** | **350** | **150** | **48** | **17k** | **3000** | **90** | **60** | **57,7** | **19,5** |
| **166** | **275** | **225** | **48** | **40k** | **1000** | **1** | **0** | **1,8** | **25,5** |
| **167** | **275** | **225** | **48** | **40k** | **1000** | **3** | **15** | **14,1** | **23,0** |
| **168** | **275** | **225** | **48** | **40k** | **1000** | **7** | **30** | **20,3** | **21,8** |
| **169** | **275** | **225** | **48** | **40k** | **1000** | **28** | **45** | **32,4** | **21,0** |
| **170** | **275** | **225** | **48** | **40k** | **1000** | **90** | **60** | **39,5** | **20,3** |
| **171** | **275** | **225** | **48** | **21k** | **2400** | **1** | **0** | **3,9** | **27,3** |
| **172** | **275** | **225** | **48** | **21k** | **2400** | **3** | **15** | **15,7** | **22,5** |
| **173** | **275** | **225** | **48** | **21k** | **2400** | **7** | **30** | **21,2** | **21,3** |
| **174** | **275** | **225** | **48** | **21k** | **2400** | **28** | **45** | **32,5** | **20,3** |
| **175** | **275** | **225** | **48** | **21k** | **2400** | **90** | **60** | **44,2** | **19,5** |
| **176** | **275** | **225** | **48** | **17k** | **3000** | **1** | **0** | **3,2** | **25,3** |
| **177** | **275** | **225** | **48** | **17k** | **3000** | **3** | **15** | **15,3** | **23,0** |
| **178** | **275** | **225** | **48** | **17k** | **3000** | **7** | **30** | **21,9** | **21,8** |
| **179** | **275** | **225** | **48** | **17k** | **3000** | **28** | **45** | **32,6** | **21,0** |
| **180** | **275** | **225** | **48** | **17k** | **3000** | **90** | **60** | **42,8** | **20,3** |
